# *Lactiplantibacillus plantarum* enables blood urate control in mice through degradation of nucleosides in gastrointestinal tract

**DOI:** 10.1186/s40168-023-01605-y

**Published:** 2023-07-19

**Authors:** Mengfan Li, Xiaoling Wu, Zewang Guo, Ruichen Gao, Zifu Ni, Hualing Cui, Minhua Zong, Filip Van Bockstaele, Wenyong Lou

**Affiliations:** 1 Lab of Applied Biocatalysis, School of Food Science and Engineering, South China University of Technology, Guangzhou, China; 2 Food Structure and Function Research Group (FSF), Department of Food Technology, Safety and Health, Faculty of Bioscience Engineering, Ghent University, Ghent, Belgium

**Keywords:** Hyperuricemia, *L. plantarum*, Gut microbiota, Network analysis, Serum uric acid

## Abstract

**Background:**

*Lactobacillus* species in gut microbiota shows great promise in alleviation of metabolic diseases. However, little is known about the molecular mechanism of how *Lactobacillus* interacts with metabolites in circulation. Here, using high nucleoside intake to induce hyperuricemia in mice, we investigated the improvement in systemic urate metabolism by oral administration of *L. plantarum* via different host pathways.

**Results:**

Gene expression analysis demonstrated that *L. plantarum* inhibited the activity of xanthine oxidase and purine nucleoside phosphorylase in liver to suppress urate synthesis. The gut microbiota composition did not dramatically change by oral administration of *L. plantarum* over 14 days, indicated by no significant difference in α and β diversities. However, multi-omic network analysis revealed that increase of *L. plantarum* and decrease of *L. johnsonii* contributed to a decrease in serum urate levels. Besides, genomic analysis and recombinant protein expression showed that three ribonucleoside hydrolases, RihA–C, in *L. plantarum* rapidly and cooperatively catalyzed the hydrolysis of nucleosides into nucleobases. Furthermore, the absorption of nucleobase by intestinal epithelial cells was less than that of nucleoside, which resulted in a reduction of urate generation, evidenced by the phenomenon that mice fed with nucleobase diet generated less serum urate than those fed with nucleoside diet over a period of 9-day gavage.

**Conclusion:**

Collectively, our work provides substantial evidence identifying the specific role of *L. plantarum* in improvement of urate circulation. We highlight the importance of the enzymes RihA–C existing in *L. plantarum* for the urate metabolism in hyperuricemia mice induced by a high-nucleoside diet. Although the direct connection between nucleobase transport and host urate levels has not been identified, the lack of nucleobase transporter in intestinal epithelial cells might be important to decrease its absorption and metabolization for urate production, leading to the decrease of serum urate in host. These findings provide important insights into urate metabolism regulation.

Video Abstract

**Supplementary Information:**

The online version contains supplementary material available at 10.1186/s40168-023-01605-y.

## Background

Hyperuricemia, characterized by high serum urate concentration over the saturation threshold (6.8 mg/dl) [1], has become one of the major public health issues which receives considerable attention [2]. In the human body, urate is the end-product of purine metabolism [3], the pivotal part of which is the last three-step degradation, where purine nucleoside is dephosphorylated to form nucleobase, followed by immediately oxidation to generate xanthine and finally to urate [2]. Previous reports claimed that the increased urate levels due to loss of uricase during hominids evolution is able to generate diverse biological functions, including radical scavenging [4], blood pressure maintenance [5], and even intelligence development [6]. However, overaccumulation of urate in the serum resulting from hepatic overproduction, renal underexcretion or their combination, has been demonstrated to be responsible for the progression of gout [1], diabetes [7], and hypertension [8]. It has been a common sense that maintaining urate homeostasis in the metabolic system in human is beneficial for health.

Various studies have demonstrated that probiotics, especially lactic acid bacteria (LAB), may impact the circulating urate levels and inflammatory mediators in vivo, thus directly or indirectly attenuating hyperuricemia [9–11]. Thereinto, the most potential hypothesis for *Lactobacilli* to decrease serum urate is inhibition of urate synthesis. Therefore, suppression of xanthine oxidase activity in liver and reduction of intestinal absorption for urate precursor are both expected to be essential for the regulation of urate synthesis [11–14]. Although a relationship exists between *Lactobacilli* of the gut and urate circulation, little has been done on the direct link between nucleoside degradation by *Lactobacilli* of the gut and serum urate levels, especially the urate synthesis in liver, based on the enzymatic, genomics, or multi-omics investigations.

This work provides substantial evidence identifying the specific role of *L. plantarum* in the reduction of serum urate in vivo. We investigated the dynamic metabolism of urate (including urate levels in serum, urine, and feces) and physiochemical parameters related with urate synthesis and excretion in hyperuricemia mice induced by a high-nucleoside diet with *L. plantarum* supplementation. We identified (i) which members of microbiota potentially contributed to the altered urate metabolism in  the host through multi-omic network analysis, (ii) the correlation between these members and *L. plantarum*, (iii) nucleoside degradation by three enzymes in *L. plantarum* towards the decrease of serum urate through genomics, in vitro recombinant enzyme activity, simulated intestinal absorption, and in vivo animal assays.

## Methods

### Mice and diets

Thirty-six and three-week-old male Kunming mice (belong to Swiss mice) were purchased from Beijing Vital River Laboratory Animal Technology Co., Ltd. All experimental procedures were approved by the Corporation of Guangdong Zhiyuan Biomedical Technology Animal Ethics Committee under protocol number IAEC-2021060402. Mice were housed at room temperature, humidity of 60–80% and specific-pathogen-free conditions on a standard 12-h light-night cycle with food and water ad libitum. The weight gain, food consumption, and amount of exercise were regularly monitored on each mouse.

A high-nucleoside diet (HNS) was prepared by combining oxonic acid (Sangon Biotech, no. A601239-0005), inosine (Macklin, no. I6219), and guanosine (Macklin, no. G6043) in a 25:37.5:37.5 ratio using 5% (w/v) sodium carboxymethylcellulose (CMC-Na). HNS group was treated with a high-nucleoside diet, which consisted of 250 mg/kg oxonic acid, 375 mg/kg inosine, and 375 mg/kg guanosine dispersing in 5% CMC-Na with total volume of 100 μL by once-daily oral gavage at 9:30 am. For HNS + LP group, mice fed with a high-nucleoside diet at 9:30 am, immediately followed by 10^7^ CFU of *Lactiplantibacillus plantarum* (LP) (derived from Chinese sauerkraut) dispersed in 100 μL saline. Twice daily oral gavage of LP was performed in which the second gavage used the same dosage of LP (10^7^ CFU) at 5 pm. For HNS + AO group, mice fed with a high-nucleoside diet at 9:30 am, immediately followed by 5 mg/kg allopurinol (AO) via once-daily oral gavage. ND mice received normal diet with equal volume of 5% (w/v) CMC-Na and saline. As for the second animal experiment, a high-nucleobase diet (HNB) consisted of hypoxanthine (Macklin, no. H6263) and guanine (Macklin, no. G810378) with 500 mM, while HNS formed by inosine and guanosine with same concentration. All mice were supplemented with oxonic acid, then half mice were fed nucleoside and another half mice were fed equal amounts of nucleobase by once-daily oral gavage at 9:30 am. Feces, urine, and eyeball serum of mice were collected every 3 days at 11:30 a.m. Urate was determined using uric acid test kit (Nanjing Jiancheng, no. C012-2–1). Mice were euthanized after 16 days of oral gavage and their tissues including intestine, kidney, and liver were collected for further evaluation, one half for histological analysis and the other half for qPCR analysis.

### DNA extraction and 16S rRNA sequencing analysis

Total DNA of microbiota was extracted from fresh fecal pellets according to the instruction of QIAamp® DNA Stool Mini Kit (Qiagen, no. 51504), then quantified using Qubit™ dsDNA HS and BR Assay Kits (Thermo Fisher, no. Q32854). The V3–V4 region of 16S rRNA was amplified using the following primers: 5′- CCTACGGGNGGCWGCAG-3′ for 341f and 5′-GACTACHVGGGTATCTAATCC-3′ for 805r. An average of 40,000 sequences in each sample were read using an Illumina Miseq™/Hiseq™ analysis and merged using PEAR-Paired-End reAd mergeR approach. Subsequently, nucleobases at the end of each read were removed if the quality score is below 20. High-quality sequences were identified to OTUs with 97% sequence similarity using UCLUST and Greengenes database 13.8. The taxonomy assignment was performed using GTDB database to blast 16S rRNA gene sequence to classify species that have both sequence similarity and coverage over 90%. The alpha diversity of gut microbiota including Chao, Ace, and Shannon index was calculated using Mothur. Principal component analysis (PCA) reflected the beta diversity of gut microbiota was carried out using R packages and GraphPad Prism software (version 8).

### Genome sequencing analysis

Sequence of *L. plantarum* obtained from Illumina Hiseq™ was transformed to reads through base calling of CASAVA. The quality of sequenced reads was further monitored and visualized by FastQC software. Then the second-generation sequences were corrected and assembled using SPAdes software. Gene counts were predicted using CRISPR analysis, and gene function was annotated according to the KEGG, COG database.

### Real-time quantitative RT-PCR analysis

The total RNA in *L. plantarum*, Caco-2 cell monolayer, and tissue of mice were extracted according to the manufacturer’s instruction (Sangon Biotech, no. SK1322). RNA concentration was determined using a micro-spectrophotometer (Merinton, SMA4000). Then 2000 ng of RNA was reverse transcribed by mixing with maxima reverse transcriptase (Thermo Scientific, 200U, no. EP0743) and 100 pmol of random primer p(dN)_6_ together to PCR analysis with 25, 50, and 85℃ for 10, 30, and 5 min, respectively. The harvested cDNA was diluted 10 times to serve as template, which is then mixed with SybrGreen qPCR Master Mix. The real-time PCR was performed using a LightCycler480 II fluorescence ration PCR instrument (Roche), and specific primers for each gene are designed by Primer Premier 5.0 software (Table S1). Finally, relative quantitation of mRNA was calculated by the CT value of reference and target gene, presented as 2^−(∆∆Ct)^, the fold change of target gene expression in a target sample relative to a reference sample, normalized to a reference gene [15].

### Histology analysis

Fresh tissues of mice including liver, kidney, and intestine were immobilized using 4% (v/v) paraformaldehyde overnight at 4 ℃. According to incremental method, enhancement concentration of ethanol was added for dehydration, then embedded in liquid paraffin. Paraffin section of 5–8 mm was then prepared from the paraffin-embedded tissues. The deparaffinized section via xylene-ethanol soaking was stained with hematoxylin–eosin solution, followed by observation on an Eclipse Ci-L, Nikon microscope.

### Biochemical analysis

Catalytic activity of xanthine oxidase (XOD) and purine nucleoside phosphorylase (PNP), concentration of H_2_O_2_, Cr, and Bun were detected using corresponding kit purchased from Nanjing Jiancheng Bioengineering Institute (No. A002-1–1, A064-1–1, C011-2–1 and C013-2–1) and Beyotime Biotechnology (No. P0321S).

### NMR analysis

Fresh fecal samples were directly collected from mice. The fecal metabolites were extracted using 100 mM potassium phosphate buffer containing 90% deuteroxide, in which 1 mM sodium 2,2-dimethyl-2-silapentane-5-sulphonate as chemical-shift reference substance ($\delta$ = 0.0 p.p.m.). Then the supernatant was analyzed using ^1^H-NMR and ^1^H–^13^C correlation NMR spectroscopy (Bluker Advance III-HD 600). Spectra was subdivided to sequential 0.02 p.p.m. Integration for each region was performed and normalized them to the total of all resonance integral regions [16, 17]. Metabolite annotations were performed according to the Human Fecal Metabolome Database (https://fecalmetabolome.ca/metabolites).

### Network analysis

Co-occurrence network construction was carried out. Abundance of all pairs of microbial operational taxonomic units (OTUs) in each group exhibited significant difference (*p* value < 0.05), which were selected as nodes. At the same time, Spearman rank correlations were calculated within OTUs. The edges would be retained once the correlation coefficient absolute value was over 0.8 in each group of mice. Therefore, the network was generated and visualized in Cytoscape [18], which showed the co-occurrence OTUs with significant change in each group (e.g., ND mice vs. HNS mice, HNS mice vs. HNS + LP mice). Also, the network parameters including degree in nodes, betweenness, and subgraph in edges were positively correlative with the contribution for change in the gut microbiota.

Multi-omic network construction was also carried out. Pearson rank correlations were calculated between OTUs, genes expression, serum urate, and fecal metabolites. In every subnetwork, significant differences (*p* value < 0.05) between high-nucleoside diet and *L. plantarum* supplementation in mice were selected as nodes. Edges were retained if the correlation coefficient absolute value was over 0.7 in HNS vs. HNS + LP comparison. The betweenness centrality, weight degree, and eigenvector centrality index of each node was calculated using Cytoscape, then the network was generated to reveal the correlation between genes, microbiota, and metabolite omics in hyperuricemia and *L. plantarum* group. In network, nodes were colored with different omics, and the side of each node responds to the number of connections.

### Degradation of nucleoside

Suspension of *L. plantarum* at density of 1 × 10^7^ CFU/mL was centrifugated to remove MRS broth and washed twice using sterile water, which is resuspended by 750 µL sodium phosphate buffer (0.1 M, pH 7.4) containing inosine (1.3 mM) and guanosine (1.3 mM). After 1-h reaction, equal volume of 5% (v/v) trifluoroacetic acid was added to terminate the degrading reaction and the mixture was filtered by a 0.22-µm membrane filter for further quantification by high-performance liquid chromatography (HPLC).

### Gene expression in E.coli DH5α

*L. plantarum* cultures (OD_600_ of 0.5–0.7) were centrifugated and resuspended in sterile water, then incubated in a 15-min boiling water bath for DNA extraction. Primer sequences of gene RihA–C are listed in Table S1, where restriction site of *BamH*I and *Hind*III were introduced at 5′ and 3′ ends, respectively. PCR reaction (Eppendorf, AG 22331) was performed for gene cloning and then the homologous recombination between PCR product and plasmid pET-28a was conducted as described using the ClonExpress® Ultra One Step Cloning Kit (Vazyme). The reconstructed plasmid was inserted into Trans5α chemically competent cell (Transgen, CD201) through chemical conversion. *E.coli* DH5α harboring reconstructed plasmid was cultured in LB medium containing 50 μg/mL of kanamycin. Once the bacteria reached mid-log phase (OD_600_$\approx$ 0.6), isopropyl-β-D-thiogalactopyranoside was added with final concentration of 0.1 µM and continued to culture at 30℃ for inducing the expression of RihA–C. After a 6-h incubation, *E.coli* DH5α cultures were centrifugated and washed three times using PBS. Then the recombinant RihA–C proteins were extracted using ultrasonication and purified using Ni-chelating affinity chromatography. Finally, concentration of proteins was detected using Coomassie brilliant blue method. The degradation rate of inosine and guanosine by RihA–C within 10 min was measured by HPLC. At the same time, RNA extraction was performed according to the instruction of UNlQ-10 Column Trizol Total RNA Isolation Kit (Sangon Biotech, no. B511321), and the following qRT-PCR analysis of RihA, RihB, and RihC mRNA levels was carried out by Sangon Biotech (Shanghai) Co., Ltd. Primer sequences of gene RihA–C are listed in Table S1.

### Cell culture, differentiation, and absorption

#### Caco-2 cell monolayer preparation

Caco-2 cells (TCHu146) were purchased from Cell Bank of the Chinese Academy of Science. In this study, Caco-2 cells grew in high-glucose DMEM (Gibco) containing 1% (v/v) of penicillin–streptomycin (Gibco) and 20% (v/v) of fetal bovine serum (Tianhang, no. 11011–8615). Caco-2 monolayer with many functions of the small intestinal villus epithelium, including food ingredient breakdown and absorption, was prepared following a protocol [19]. In detail, Caco-2 cells of 42 passage numbers were seeded in apical chamber of 12-well transwell (Corning, no. 3470) with density of 3 × 10^5^ and volume of 0.5 mL per well, and then 15 mL of growing medium was added on a basolateral chamber. Cells were incubated at 37℃ in an atmosphere of 5% CO_2_, and with pre-existing medium replaced by identical volume of fresh medium every second day during a period of 21 days.

#### Caco-2 cell monolayer differentiation analysis

After 21-day differentiation, cell monolayers were collected using a scraper, immersed in 2.5% glutaraldehyde and stored at 4℃ for 1 h for cell fixation, then washed thrice using PBS (0.1 M, pH 7.0). Subsequently, 1% osmic acid were added to further fixate cells for 2 h and dehydration was carried out using gradient increasing concentration of ethanol solution. Finally, cell monolayer was immersed in acetone for 20 min and then embedded with Epon812 for overnight. Longitudinal section of cell monolayer with thickness in range of 70–90 nm was obtained using a ultramicrotome (LEICA EM UC7), stained by lead citrate and uranyl acetate for 5–10 min. After drying in the air, the differentiated phenotype of cell monolayer was observed using a biological transmission electron microscope (TEM, HITACHI HT7800). In addition, intracellular alkaline phosphatase activity of cell monolayers on apical chamber was measured according to the manufacturer’s instructions (Beyotime, no. P0321M).

#### Caco-2 cell monolayer integrity analysis

Trans-epithelial electrical resistance (TEER) values were measured using an electrical resistance meter (Millcell ERS-2, Millipore) every 2 days. After differentiation for 21 days, the cell monolayers with TEER value at 37 ℃ over 300 Ω cm^−1^ were used for absorption experiment. In addition, top view of cell monolayers was obtained using an optical microscope. Localization and distribution of tight protein occludin and nucleus in cell monolayer were analyzed using an immunofluorescence microscopy approach [20]. In detail, cell monolayer was immersed in 4% paraformaldehyde for 30 min at room temperature. After a washing step with PBS, 0.1% Triton X-100 in PBS was added to permeabilize cell monolayer for 10 min. Then the cell monolayer was incubated in mouse anti-occludin monoclonal antibody (Thermo Fisher Scientific, 1:200, no. OC-3F10) for 90 min. Subsequently, cell monolayer was further incubated in 1 μg/mL 4*’*,6-diamidino-2-phenylindolesecondary (DAPI, Solarbio, no. D8200) and antibody Alexa594-conjugated phalloidin (Thermo Fisher Scientific, 1:500, no. A12381) for 90 min. After a washing step with PBS and dehydration with ethanol, fluorescence was detected using a confocal laser scanning microscope (CLSM, Zeiss, no. LSM780) at ex wavelengths of 405 and 543 nm. Besides, the apparent permeability coefficient (*P*_app_) of marker uranine from apical to basolateral chamber was monitored. Herein, 100 μg/mL uranine in Hank’s balanced salt solution without phenol red (HBSS) was added on apical sides of cell monolayer, after a 2-h incubation, concentration of uranine on basolateral side was detected using a microplate reader (Agilent BioTeK, Synergy Neo2) at emission wavelength of 536 nm and excitation wavelength of 724 nm.

#### Absorption of nucleosides and nucleobases

After a 21-day incubation time for cell differentiation, cell monolayers were washed thrice using prewarmed HBSS. Inosine or guanosine in 0.45 mL HBSS was added on the apical side and 1.2 mL of HBSS on the basolateral side, then incubated at 37 ℃ with 100 rpm of shaking for 2 h. A certain volume (50 μL) of mixture on apical side was sampled and replaced by same volume of HBSS every half hour. The nucleoside and nucleobase content on apical and basolateral sides were detected using HPLC. *P*_app_ (cm s^−1^) of inosine and guanosine was calculated by the absorptive concentration (µM) of nucleoside per second divided by the multiplication of surface area (cm^2^) of the apical chamber and the initial concentration (µM) of nucleoside in the apical chamber.

### LC–MS/HPLC analysis

Transformation of inosine and guanosine catalyzed by *L. plantarum* and its recombinant enzymes RihA–C were identified by LC–MS (Agilent1290/Bruker maXis impact) with HRMS model. MS analysis supported from https://hmdb.ca/metabolites/HMDB0000157#spectra. The concentration of hypoxanthine, guanine, inosine, guanosine, xanthine, and urate were further detected by HPLC (Waters 2690) equipped with a detection wavelength of 254 nm, Agilent ZORBAX SB-C18 column (4.6 × 250 mm 5-Micron) and mobile phase of 25 mM KH_2_SO_4_ buffer containing 5% (v/v) methanol.

## Results

### *L. plantarum* attenuates hyperuricemia in mice

**Fig. 1:**
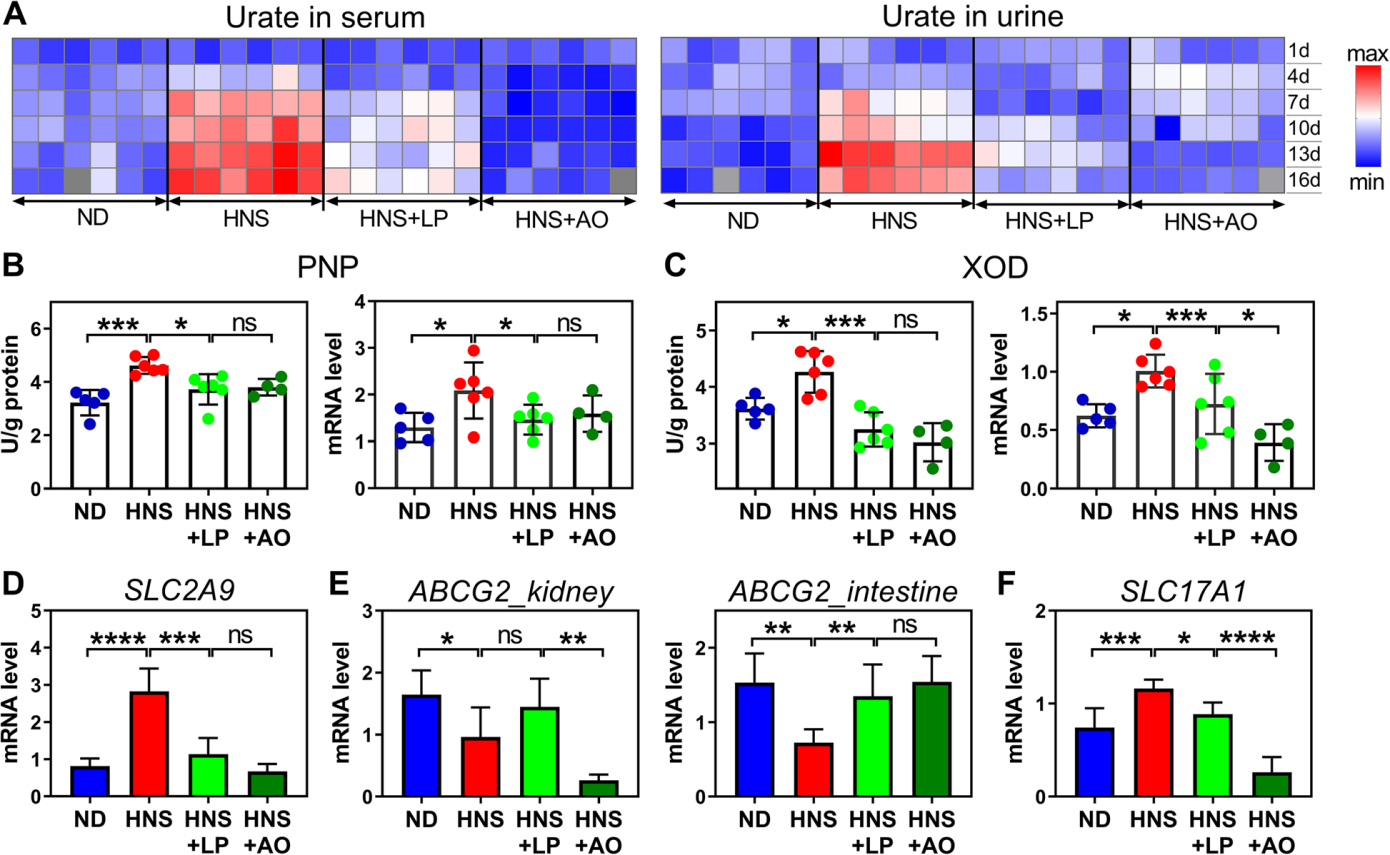
*L. plantarum* can suppress urate synthesis and improve urate excretion by intestine. **A** Heatmap showing urate level in serum and in urine of mice fed ND, HNS, HNS + LP, and HNS + AO for a consecutive 16 days. The red and blue colors show higher and lower levels of urate. Data are visible in Fig. S1A. **B–F** Synthesis and excretion of urate in mice. **B**, **C** Enzymatic activity (left panel) and corresponding mRNA level (right panel) of purine nucleoside phosphorylase (PNP) (**B**) and xanthine oxidase (XOD) (**C**) in liver of mice at 16 days after euthanasia. Activity of PNP and XOD indicated the synthesis of urate. **D–F** The qRT-PCR analysis of mRNA levels of urate transporter, including *SLC2A9* (**D**), *ABCG2* (left panel in **E**), and *SLC17A1* (**F**) involved in urate excretion by kidney. Right panel in **E** shows mRNA level of *ABCG2* associated with urate excretion by intestine. ND, normal diet; HNS, high-nucleoside diet; HNS + LP, high-nucleoside diet supplemented with *L. plantarum*; HNS + AO, high-nucleoside diet plus allopurinol treatment; *n* = 6 mice per group unless otherwise stated. Graphs show the mean (**A**) and mean ± s.d. (**B–F**); *P < 0.05, **P < 0.01, ***P < 0.001, ****P < 0.0001 by two-tailed* t*-test (**B**, **C**) and one-way ANOVA with *t*-test correction (**D**–**F**)

We started by inducing hyperuricemia in Kunming mice by feeding them with high-nucleoside diet (HNS) appending a uricase inhibitor oxonic acid, to yield murine phenotypes that mimic human hyperuricemia [13, 21, 22]. As expected, when compared with mice receiving a normal diet (ND), the mice fed with HNS exhibited high levels of serum urate (Fig. 1A, Fig. S1A). However, no phenotypic changes (e.g., body weight, weight ratio of kidney, and liver to body weight) were observed among them (Fig. S1B, C). To identify potential differences in circulating urate with/without supplementation of *L. plantarum* in HNS mice, the urate level in serum, urine, and feces was monitored after gavage with 10^7^ colony-forming units (CFU) of *L. plantarum* twice daily over consecutive 16 days. Interestingly, when compared with HNS mice, the mice supplemented by *L. plantarum* (HNS + LP) exhibited lower serum urate levels on the 4th day and thereafter (Fig. 1A, Fig. S1A), with tendency similar to the group of allopurinol treatment (HNS + AO), a urate-lowering therapy [23]. However, mice given allopurinol at dose of 5 mg/kg per day led to renal injury, as indicated by the increased weight ratio of kidney to body weight (Fig. S1C), elevated renal function indexes of creatinine (Cr), and urea nitrogen (Bun) (Fig. S1D), and visible crystals in H&E-stained images (Fig. S1E), which were not observed in the HNS + LP mice. Moreover, the pathological changes of liver tissue caused by a high-nucleoside diet were ameliorated after *L. plantarum* supplementation, evidenced by the many inflammatory cell infiltrations in HNS mice and few in HNS + LP mice (Fig. S1E). These results suggested the positive role and safety of *Lactobacilli* supplementation for reduction of serum urate levels.

To assess the effects of *L. plantarum* on urate metabolism, we first performed activity detection and qRT-PCR analysis of the key enzymes that were involved with urate synthesis in the liver of mice. Purine nucleoside phosphorylase (PNP) participates in the purine metabolism by converting nucleoside to nucleobase, e.g., inosine to hypoxanthine and guanosine to guanine. High nucleoside intake led to enhancement of PNP activity and corresponding mRNA level (Fig. 1B), which suggested that oral nucleoside is able to cross the intestinal barrier, enter the blood circulation, and follow the degradation catalyzed by hepatic PNP. However, this pathway was suppressed by *L. plantarum*, as evidenced by the significant change in PNP activity between HNS and HNS + LP mice (Fig. 1B). Xanthine oxidase (XOD) is a critical, rate-limiting enzyme that manages sequential oxidative hydroxylation of hypoxanthine to xanthine and to urate, and generates hydrogen peroxide (H_2_O_2_) [2]. Similar to PNP, XOD in HNS mice displayed remarkably higher activity and expression level than those in ND and HNS + LP mice (Fig. 1C, Fig. S1F). Consequently, overaccumulation of urate and H_2_O_2_ in HNS mice was observed (Fig. 1A, Fig. S1G), while ND and HNS + LP mice displayed lower levels of both substances. Similar tendency was observed in HNS + AO mice as HNS + LP mice (Fig. 1B, C and Fig. S1G). These results provide evidence that urate overproduction is effectively suppressed by *L. plantarum* and allopurinol treatment, which might be involved with the decrease in enzymatic activities of XOD and PNP in liver.

The expression of urate transporter genes was measured to confirm the effect of *L. plantarum* on urate excretion in kidney and gastrointestinal tract of mice. The urate transporter genes *SLC2A9* and *SLC22A12* are responsible for the reabsorption of urate in the basolateral and apical membrane of proximal renal tubules [24, 25]. Notably, one of the genes, *SLC2A9,* was upregulated in the kidney of HNS mice as compared with the ND mice (Fig. 1D, Fig. S1H). *ABCG2* gene, which is involved in urate excretion in renal and intestinal transport [26] showed significant downregulation in HNS mice (Fig. 1E). Moreover, another gene, *SLC17A1,* which is strongly positive correlated with urate excretion merely in kidney [27], displayed a significantly increased mRNA level after a high-nucleoside diet (Fig. 1F). Although the contribution of aforementioned genes toward serum urate far outweighs that of other genes [28], the quantitative contribution of specific gene to renal excretion of urate still remains ambiguous. In this study, the opposite regulatory effect between *ABCG2* gene and *SLC17A1* gene and the increased urate in urine from HNS mice (Fig. 1A, Fig. S1A) implied the predominant role of *SLC17A1* gene in renal excretion of urate. These results suggest that elevated serum urate in HNS mice resulted from the following two pathways: (i) strengthened urate synthesis mediated by XOD and PNP enzyme, and (ii) suppressed urate excretion through gastrointestinal tract potentially mediated by *ABCG2* gene. Interestingly, the deteriorated urate metabolism due to HNS diet was ameliorated after *L. plantarum* supplementation. Regarding HNS + AO mice, the behavior of expression of renal *SLC2A9* and intestinal *ABCG2* gene were similar to that of HNS + LP and ND mice. However, significant differences of both renal genes (*ABCG2* and *SCL17A1*) between HNS + AO and ND mice suggested the abnormal urate excretion in kidney. Obviously, allopurinol treatment might have an impact on the renal function involved with urate excretion, although the significant decrease in serum urate was observed (Fig. 1A).

### *L. plantarum* affects gut microbiota and metabolites

Besides identifying the effect of *L. plantarum* on urate metabolism for hyperuricemia, it is critical to build the relationship between hyperuricemia and gut microbiota. First, to reveal the effects of a high-nucleoside diet and *L. plantarum* supplementation on the composition of gut microbiome, we performed the 16S rDNA gene sequencing to analyze the feces from mice. The over 30,000 reads of each sample in sequencing depth demonstrated the saturated detection for intestinal bacteria (Fig. S2A). Analysis of alpha diversity of microbial community (e.g., Shanon index) between ND and HNS mice, HNS and HNS + LP mice did not display any significant differences (Fig. S2B). However, significant changes in beta diversity of microbiota were observed in HNS mice in comparison with ND mice, as indicated by the increased *Firmicutes/Bacteroidetes* ratio (Fig. S2C) and the separate zone in principal component analysis (PCA) based on microbiota abundance at the phylum level (Fig. S2D). Nevertheless, no significant change in HNS + LP mice is detected when compared to HNS mice. These results are consistent with previous publications showing that probiotics administration did not significantly change the fecal microbiota composition [29, 30]. Further comparison on operational taxonomic units (OTUs) was performed to better distinguish the difference among microbiomes. Co-occurrence network contained 172 edges between 39 nodes (microbial OTUs) (Fig. S2E), suggesting that HNS diet and *L. plantarum* supplementation gave rise to the changes in microbial abundances. Especially, fifteen differential genus pointed to the significant differences in the microbiota of HNS and HNS + LP mice (Table S2).

**Fig. 2:**
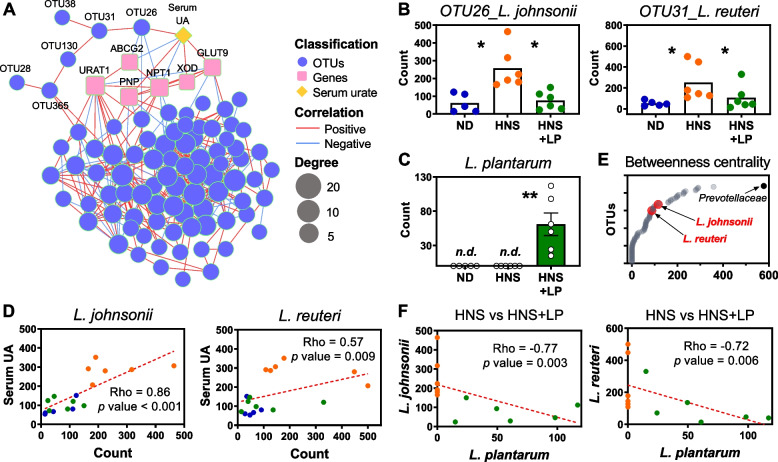
*L. plantarum* decreases *L. johnsonii* and *L. reuteri* in gut. **A** Multi-omic network integrating microbial abundances (blue circles) with serum urate level (yellow diamond) and gene expression of key genes (pink squares) involved in urate synthesis and excretion. Node size and green outline of nodes correspond to degree (number) that combined with other nodes. Red and blue edges represent positive and negative correlation, respectively, between two nodes. Edge (line) weight is proportional to correlation coefficient. Data are visible in Tables S9 and S10. **B**, **C** Abundance of two *Lactobacillus* species including *L. johnsonii* (left panel in **B**) and *L. reuteri* (right panel in **B**) and *L. plantarum* (**C**) in feces of mice fed ND, HNS, and HNS + LP. Graphs show the mean ± s.e.m. Each symbol represents a mouse, and *n.d.* means not detected. *P < 0.05, **P < 0.01 by one-tailed *t*-test. **D** Scatter plot for correlation analysis between ND (blue), HNS (orange), and HNS + LP mice (green). Dots indicate the abundance of microbe (*X* axis) versus serum urate level (*Y* axis). **E** Betweenness centrality responds to the importance for altering gut microbiota and urate metabolism. Every symbol represents one operational taxonomic unit (OTU). **F** Scatter plot for correlation analysis between HNS (orange) and HNS + LP mice (green). Dots indicate the abundance of *L. plantarum* versus other microbes. The dotted line shows the fitted line. Graphs show the Spearman rho correlation coefficient and *p* value

To identify potential microbes that accounted for urate metabolism regulation, we performed the multi-omic network analysis integrating microbial abundances with metabolic parameters regulated by *L. plantarum* supplementation (Fig. 2A). The network contained 342 edges connecting 97 nodes. Notably, a branch of microbe nodes (OUTs) was directly connected to serum urate, which was significantly decreased after *L. plantarum* supplementation. Next, detection of the taxonomy of these microbes using NCBI Nucleotide BLAST analysis (Table S3) showed that four OTUs significantly aligned with four bacterial species, *Lactobacillus johnsonii*, *Lactobacillus reuteri*, *Lactobacillus murinus*, and *Lactobacillus intestinalis*. Compared to HNS mice, HNS + LP mice remarkedly decreased abundance of *Lactobacillus johnsonii* and *Lactobacillus reuteri* (Fig. 2B), but slightly increased abundance of *Lactobacillus murinus* (Fig. S2F). As expected, *L. plantarum* is detected in feces of HNS + LP mice (Fig. 2C). Thus, *L. plantarum* was suggested to play a key role in regulating these *Lactobacillus* species, which may affect urate metabolism in the host. Our inference is further substantiated by the correlations between serum urate level and the abundance of both *Lactobacillus johnsonii* and *Lactobacillus reuteri* species in host (Fig. 2D, Fig. S2G, H), which has been previously validated to be associated with type 2 diabetes in mice and humans [31, 32]. Furthermore, these two *Lactobacilli* were negatively correlated with *L. plantarum* (Fig. 2F, Fig. S2I). The results revealed that *L. plantarum* contributed to a lower abundance of *Lactobacillus johnsonii* and *Lactobacillus reuteri*, which were positively associated with serum urate in hyperuricemia mice.

Betweenness centrality is characterized by the frequency with which a node connects other nodes within a network, suggesting the importance to altering gut microbiota and serum urate. Therefore, we examined whether these *Lactobacillus* in network are among microbes that significantly affect host urate metabolism. The *Lactobacillus* predicted to influence the metabolic parameters in host had a high value, but not the highest (Fig. 2E), suggesting that microbes with potentially high effect on the serum urate in host may not necessarily play a central role in modulating the gut microbial community. In contrast, *Prevotellaceae* with the highest betweenness centrality did not show the direct connection with serum urate (Fig. 2A). Several studies have identified the intestinal *Prevotellaceae* interacts with Parkinson’s disease [33], obesity [34], and constipation [35]. Furthermore, the direct association between abundance of *Prevotellaceae* and some toxic molecules in serum has been found in patients with end stage renal disease, however, these toxic molecules are indoxyl sulfate, p-cresol, and C-reactive protein, rather than serum urate [36]. The relevance of *Prevotellaceae* with regard to serum urate remains unclear, although their association was slightly suggested in this study.

In addition, using ^1^H NMR and ^1^H-^13^C correlation NMR analysis [16], we detected the 35 fecal metabolites (Fig. S3A), wherein the twenty substances were elevated in HNS + LP mice (Fig. S3B). In order to identify the potential metabolites responsible for decreased serum urate, multi-omic network analysis was performed (Fig. S3C) and showed that serum urate was predominantly connected to eight metabolites, which were significantly increased by *L. plantarum* supplementation. Of which, short-chain fatty acids (SCFA) and glucose are known as energy providers for urate excretion transported by *ABCG2* in intestine [37]. Conversely, the drop in these metabolites might be explained by the phenomenon that intestinal excretion of urate decreased in HNS mice (Fig. S1A). Besides, these elevated metabolites were closely connected to *Lachnospiraceae* and *L. johnsonii*, with the former increased and the latter decreased in HNS + LP mice*.* Especially, *Lachnospiraceae* is known to be involved in the generation of SCFA such as propionate [38]. Though previous studies suggested that gut microbiota and its metabolites are involved in the progression of hyperuricemia [39], the precise signs corresponding to hyperuricemia are not yet clear. Here the decreased abundance of *L. johnsonii* and increased concentration of eight metabolites is possibly the gut-modulated pathway used by *L. plantarum* to decrease serum urate in hyperuricemia mice.

### Three isoenzymes in *L. plantarum* transform exogenous nucleoside into nucleobases

**Fig. 3:**
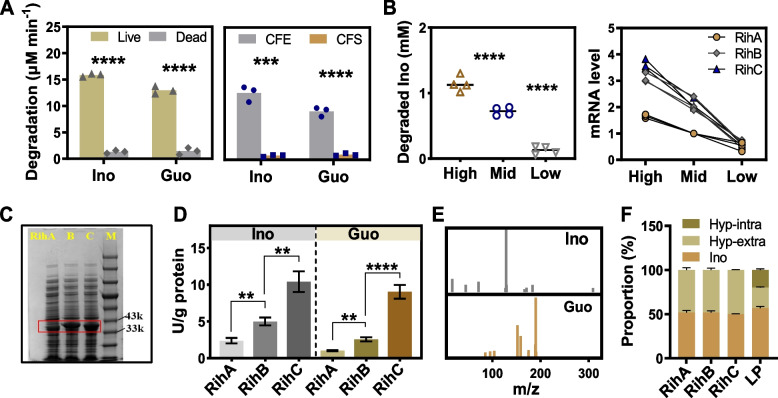
*L. plantarum* transforms exogenous nucleoside into nucleobase. **A** Degradation rate of inosine (Ino) and guanosine (Guo) by live or dead *L. plantarum* (left panel), by intracellular cell-free extract (CFE) or cell-free supernatant (CFS) of *L. plantarum* (right panel).** B** Degradation ability of live *L. plantarum* (left panel) and mRNA levels (right panel) of key enzymes RihA, RihB, and RihC after administration of different amounts of inosine (concentration of 1.14 ± 0.12 mM (High), 0.72 ± 0.06 mM (Middle), and 0.13 ± 0.06 mM (Low)). **C** SDS-PAGE showed the molecular weight of recombinant enzyme Rih A–C in *E. coli* BL21 (DE3) after induced expression. M refers to marker. **D** Enzymatic activity of recombinant RihA, RihB, and RihC towards hydrolysis of inosine and guanosine. **E** Mass spectra confirmed the degradation product of inosine and guanosine to be hypoxanthine and guanine, respectively. **F** Proportion of substrate inosine and product hypoxanthine after 1 h reaction catalyzed by Rih A–C and live *L. plantarum*. Hyp-intra and hyp-extra refers hypoxanthine (Hyp) detected intracellularly and extracellularly, respectively. Graphs show the mean (**A**, **B**) and mean ± s.d. (**D**, **F**). Each data point represents three independent biological replicates (**A**, **B**). Data was analyzed by two-tailed *t*-test (**A**, **D**) and one-way ANOVA with *t*-test correction (**B**). **P < 0.01, ***P < 0.001, ****P < 0.0001

Despite the important role of alteration of gut microbiota and its metabolites on the control of urate metabolism, the above results still did not exclude the possibility of the intrinsic function of *L. plantarum* itself towards the decrease of serum urate. Therefore, we performed the whole genome sequencing analysis and annotated gene functions in *L. plantarum*. We first focused on the function of nucleotide transport and metabolism in *L. plantarum*, which matched 92 and 143 genes in COG and KEGG orthology, respectively (Fig. S4). Three genes (PROKKA_00051, PROKKA_01337 and PROKKA_00511) annotated as ribonucleoside hydrolase (RihA, RihB and RihC) (Table S4), may be involved in alteration of urate metabolism in HNS + LP mice. To validate our assumption, we confirmed the degradation of nucleoside inosine and guanosine by live whole-cell and intracellular cell-free extract (CFE) of *L. plantarum* (Fig. 3A), whereas dead cell and cell-free supernatant (CFS) did not. Moreover, the mRNA levels of ribonucleoside hydrolases were positively correlated with nucleoside degradation ability in *L. plantarum* (Fig. 3B). The results suggested that these genes may be involved in the nucleoside degradation in *L. plantarum*. To further verify the nucleoside hydrolyzing ability of these genes, we cloned them from *L. plantarum* and obtained the enzyme from recombinant *E. coli* BL21 (DE3), which showed molecular weight located in the range of 33–43 kDa (Fig. 3C). Specifically, the recombinant hydrolases displayed different catalytic activity, with RihC having the highest activity, followed by RihB and RihA (Fig. 3D), which suggested that these genes/proteins are probably critical for *L. plantarum* to perform the efficient degradation of nucleoside. Next, the hydrolysis product of inosine and guanosine was examined to be typical nucleobase, hypoxanthine and guanine, respectively (Fig. 3E, Fig. S5). In addition, concurrent decrease of substrate nucleosides and increase of product nucleobases with proportional relationship is detected after degradation reaction by *L. plantarum* or by the recombinant hydrolases (Fig. 3F). Despite the similar activity of RihA, B, and C in *L. plantarum* with PNP in the host to catalyze the conversion of inosine and guanosine, the generated hydrolysis products hypoxanthine and guanine fail to serve as substrates for urate synthesis. No enzyme in *L. plantarum* is detected to be able to catalyze the transformation of guanine and hypoxanthine into xanthine and resultant urate (Table S5). Hence, guanine and hypoxanthine were available for uptake by intestine in HNS + LP mice, which is different from the case of HNS mice where inosine and guanosine were available. Furthermore, *L. plantarum* can also uptake a certain amount of nucleobases transported by guanine/hypoxanthine permease PbuG (Table S6), evidenced by the detected intracellular hypoxanthine and guanine in *L. plantarum* (Table S7). The simultaneous conversion of nucleosides and uptake of nucleobases by *L. plantarum* might decrease their intestinal absorption in host, consequently decelerating the production of urate.

### Limited absorption of nucleobases leads to remarkably less urate synthesis than nucleosides

**Fig. 4:**
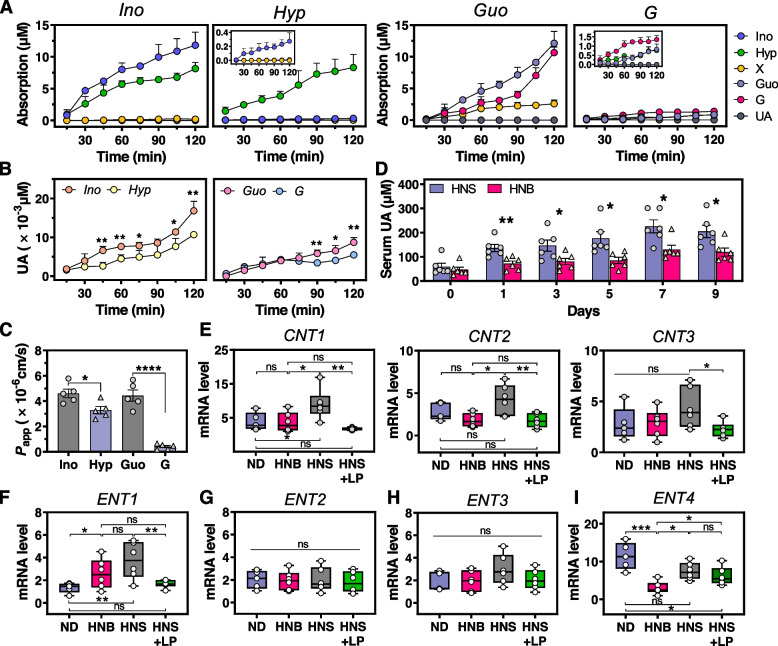
Absorption and metabolism of nucleosides and their constituent nucleobases. Concentration changes of various compounds (**A**) and urate (**B**) on basolateral side when Caco-2 cell monolayer absorbed nucleosides (inosine and guanosine) and nucleobases (hypoxanthine and guanine) from apical side. The apical and basolateral side of Caco-2 cell monolayer is considered as the bowel luminal and blood side, respectively. Ino, inosine; Hyp, hypoxanthine; X, xanthine; Guo, guanosine; G, guanine; UA, urate. **C** Apparent permeability coefficient of nucleosides and nucleobases in Caco-2 cell monolayer. **D** Serum urate in mice fed by nucleosides (HNS) and nucleobases (HNB), respectively. *n* = 6 mice per group. RT-PCR analysis of mRNA levels of genes CNT1–3 (**E**) and ENT1–4 (**F**–**I**), which are involved with transport of nucleosides and nucleobases in the intestine of mice. ND, normal diet; HNB, nucleobase diet; HNS, nucleoside diet; HNS + LP, nucleoside diet with *L. plantarum* supplementation. Each data point represents three independent biological replicates. Graphs show the mean ± s.e.m. (**A**–**D**) and median with min to max (**E**–**I**). Data was analyzed by one-tailed (**A**, **B**, **D**, **F**) and two-tailed (**C**, **E**, **G**, **H**, **I**) *t*-test. *P < 0.05, **P < 0.01, ***P < 0.001, ****P < 0.0001

The remarkably higher solubility of inosine and guanosine in water than its constituent nucleobase (Table S8) drew our attention to whether the intestinal absorption rate for nucleosides was higher than that of nucleobases. First, Caco-2 cell monolayer was established for mimicking human intestinal epithelial layer to predict permeability and absorption of oral food ingredient [19]. A differentiated phenotype with microvilli and a tight junction between cells (Fig. S6A–C) and enhanced activity of intracellular alkaline phosphatase (AKP) (Fig. S6D) were observed, suggesting that the Caco-2 cell monolayer featured many functions of the small intestinal villus epithelium, including food ingredient breakdown and absorption [40]. Additionally, the trans-epithelial electrical resistance (TEER) value of over 300 Ω cm^2^ (Fig. S6E) and decreased paracellular permeability of marker uranine (Fig. S6F) indicated the integrity of the obtained Caco-2 monolayer. It is acknowledged that both hepatic and intestinal epithelium cells can generate urate due to the presence of a series of intracellular oxidases, including PNP and XOD. To identify the effect of nucleosides and their constituent nucleobases on the level of serum urate, we monitored the changes of these purine metabolites within the 2 h absorption of differentiated Caco-2 cell monolayer. The concentration of absorbed inosine and guanosine were remarkedly higher than those of hypoxanthine and guanine, respectively (Fig. 4A). Interestingly, more basolateral urate was generated after intestinal absorption of nucleosides (Fig. 4B). Apparent permeability coefficient (*P*_app_) of inosine and guanosine, which signified intestinal absorption rate of oral compound [40], was significantly higher than their constituent nucleobases (Fig. 4C). To determine whether these results are also presented in vivo, mice were gavaged with same number moles of nucleosides and nucleobases, respectively, and serum urate was detected 2 h later. Mice fed with nucleosides generated more urate in serum than that by nucleobases (Fig. 4D), and this phenomenon lasted over a period of 9-day gavage.

Although extensive studies reported the association of *SLC28* (coded by CNT1–3) and *SLC29* family proteins (coded by ENT1–4) with transport of nucleoside and nucleobase substances [41, 42], the substrate selectivity of each protein was still obscure [43]. Therefore, we performed qRT-PCR analysis of these two groups of transporters in intestinal epithelial cells of mice. In apical membranes of intestine, CNT1, CNT2, and CNT3 would be capable of transporting nucleoside, indicated by the significant increase of their mRNA levels (Fig. 4E). In basolateral membranes, ENT1 is able to transport nucleoside and nucleobase (Fig. 4F), in agreement with previous studies [44, 45]. No significant difference was observed in mRNA levels of ENT2, ENT3, and ENT4 between mice fed with high-nucleoside and nucleobase diet (Fig. 4G–I) whereby ENT4 previously identified as adenosine-specific transporter [46]. Conversely, mRNA level of ENT4 seemed to be inhibited by both nucleoside and nucleobase (Fig. 4I), which might be affected by environmental pH due to pH-dependent activity of ENT4 which exhibited almost no transport activity at pH 7.4. Overall, these results agreed with our assumption that nucleoside exhibited higher intestinal absorption rate and generated more urate than their constituent nucleobase, which might be ascribed to the presence of more transporters for nucleoside. It is noteworthy that similar behavior of these transporters in HNB and HNS + LP mice was observed (Fig. 4E–H). The results provide evidence that the blood urate control by *L. plantarum* can be attributed to its capability of degrading nucleosides into nucleobases.

## Discussion

Comprehensive and systematic data on the global prevalence of hyperuricemia was reported in the range 7‒32% in various ethnicities, ages, genders, and locations, while that of gout ranged from less than 0.5% to as high as 10.3% [47–49]. Studies from USA and Australia showed that the prevalence of both gout and hyperuricemia has stabilized [50, 51]. However, recent estimates indicate that the prevalence and incidence continue to rise in most countries, including developed and developing countries [52]. Obviously, hyperuricemia and gout are common diseases and present a global problem for health care systems. There is no doubt that the increasing prevalence of gout and hyperuricemia propel the cost of urate-lowering and corresponding analgesic therapies [53]. And another bigger concern is the occurrence of a serious adverse effect due to the drug treatments, e.g., subclinical hypothyroidism [54] and kidney function impairment [55] induced by administration of the urate-lowering medicine allopurinol, an increased risk of cardiovascular death caused by supplementation of another urate-lowering medicine febuxostat [56]. Probiotics, especially the LAB, have been widely used in the manufacture of dairy products such as yogurt, cheese, and pickled vegetables. Increasing evidence underscores the beneficial effects of the lactic acid bacteria on human physiology and pathology. Among the most distinctive benefits of *Lactobacillus* is protection against chronic disease hyperuricemia [12–14].

Several publications have reported the phenomenon *Lactobacillus* spontaneously absorb the extracellular nucleosides or nucleobases [13, 22], importantly, it has been linked to urate levels in the circulation. On the basis of this, yogurt with living *Lactobacillus* which was pronounced to have urate-lowering function, is produced and sold on Japanese markets [57]. It is clear that the ability to absorb the nucleosides by *Lactobacillus* plays an important role in host urate metabolism. Nevertheless, the molecular mechanisms underlying these effects have barely been studied. This work reveals that *L. plantarum* suppresses the intestinal absorption of urate precursor and metabolism, which regulates urate synthesis in the liver in response to oral intake of nucleosides. *L. plantarum* isolated from Chinese pickle exhibited the ability to degrade the nucleosides including guanosine and inosine. Using hyperuricemia mice induced by high-nucleoside diet, we have identified the role of *L. plantarum* in inhibiting the activity of hepatic XOD and PNP for urate synthesis (Fig. 1B, C). Although several in vivo studies showed the suppression in XOD activity by probiotics [11, 12], we observed the reduction levels of PNP activity in mice supplemented with *L. plantarum*, suggesting the unique role of *L. plantarum* in nucleoside management in gastrointestinal tract. Further analyses of genomics and recombinant enzyme activity assays revealed that *L. plantarum* featured nucleoside degradation ability depending on its three intracellular hydrolase RihA, RihB, and RihC (Fig. 3). Previous study indicated the inosine hydrolyzing ability of *L. brevis* DM9218 is contributed by an intracellular enzyme ORF00084 [14], which has 84% homology in nucleotide sequence when compared with that of RihA (Fig. S7). RihB has high similarities with another *Lactiplantibacillus plantarum* species with 88% homology, and 85% for RihC with both *Levilactobacillus brevis* and *Limosilactobacillus fermentum*. Obviously, nucleoside hydrolase is widely present in *Lactobacillus* species, even in other organisms, e.g., *E. coli.* (SQP21335), *Clostridium* (EXG88370), *Acidobacteria* (TDI55063) and so on. However, the impact of these nucleoside hydrolases on the host serum urate is rarely studied. We disclose one important new mechanism used by *L. plantarum* which catalyzes the efficient nucleoside degradation to enable the control of urate metabolism in the host. Degradation of extracellular nucleoside to nucleobase performed by three ribonucleoside hydrolases RihA–C (Fig. 3A) and accompanying uptake of nucleobase of *L. plantarum* (Table S7) together might decrease the concentration of urate precursors in the gastrointestinal tract. Furthermore, the presence of fewer nucleobase-specific transporters in intestinal epithelial layer might be important in the case of poor absorption of nucleobase. The decreased content of nucleosides and the limited absorption of converted nucleobases by intestine altogether might have impacts on the decreased expression of PNP and XOD and their enzymatic activity (Fig. 1B, C), and resultant lower urate concentration in blood (Fig. 4D–F).

Multi-omic network analysis suggests the possibility of microbe-microbe interaction and their metabolites working on urate metabolism in *L. plantarum* supplemented mice (Fig. 2A). Specifically, *L. plantarum* decreased the abundance of *L. johnsonii*, which directly responded to serum urate. Additionally, we observed eight metabolites that were elevated in response to *L. plantarum* supplementation, which may contribute to the bacterium’s effect on lowering serum urate in the host (Fig. S3C). Although *L. plantarum* is not dominant bacteria in abundance in gut microbiota of HN + LP mice, serum urate decreased by supplementation of single bacteria does still not exclude the possibility of microbe-microbe interaction and their metabolites working. Here, *L. plantarum* is negatively correlated with *L. johnsonii*, variations in abundance of which directly responded for serum urate according to the multi-omic network analysis (Fig. 2A, F). A recent study has demonstrated the vital role of *L. johnsonii* in improving glucose metabolism and thus attenuating of western diet-induced diabetes [31]. In this work, we found the similar interaction between abundance of *L. johnsonii* and glucose levels (Fig. S3C); however, its effects on purine metabolism and altered serum urate need to be further investigated.

## Conclusion

In conclusion, our study points to the possible mechanism at the molecular level that oral administration of single microbe, *L. plantarum,* is beneficial for reduction of serum urate via its power to transform nucleoside into nucleobase, which partially block the synthetic pathway of urate. However, it is crucial that the pathway might be effective in hyperuricemia mice caused by the dietary induction, but not in hyperuricemia mice induced by the genetic or intrinsic factors. This study is anticipated to provide insights into understanding of hyperuricemia metabolism in mice but does not yet make clear if it is consistent with hyperuricemia that occurs in humans.

## Supplementary Information

**Additional file 1: Table S1.** Primer sequence of genes in this work. **Table S2.** Fifteen differential genera in mice supplemented *L. plantarum* when compared with HNS mice. **Table S3.** Taxonomy of six microbes using NCBI Nucleotide BLAST analysis tool. **Table S4.** Three genes involved with nucleoside degradation in *L. plantarum*. **Table S5.** Annotated genes contributed to the nucleotide and nucleoside metabolism, which showed that no enzyme in *L. plantarum* enable to catalyze the transformation of guanine and hypoxanthine into xanthine and resultant urate. **Table S6.** Genes involved with purine permeation in *L. plantarum*. **Table S7.** Permeation for nucleobases in *L. plantarum* within 2 hours. **Table S8.** Solubility of nucleosides and nucleobases in water. **Table S9.** Nodes information in multi-omic network analysis given by Fig. 2A. **Table S10.** Edges information in multi-omic network analysis given by Fig. 2A. **Table S11.** Nodes information in multi-omic network analysis given by Fig. S3C.** Table S12.** Edges information in multi-omic network analysis given by Fig. 3C.

**Additional file 2: Figure S1.** Changes in metabolic parameters due to high nucleosides diet and *L. plantarum* supplementation in mice. **Supplementary Fig. 2.** Changes in gut microbiota due to high nucleosides diet and *L. plantarum* supplementation in mice. **Supplementary Fig. 3. ***L. plantarum* affecting the gut metabolites. **Supplementary Fig. 4.** Metagenomic functional annotation and classification of *L. plantarum* in KEGG (A) and in COG (B).** Supplementary Fig. 5.** Identification of production of inosine hydrolyzed by* L. plantarum*.** Supplementary Fig. 6.** Established Caco-2 cell monolayer for nucleosides and nucleobases transport. **Supplementary Fig. 7.** Gene sequences alignment of RihA–C from *L. plantarum* with other organisms.

## Data Availability

The 16S rRNA gene sequence of gut microbiota, genome sequence of *Lactiplantibacillus plantarum* and its RihA–C enzymes generated in this study have been deposited in the National Center for Biotechnology Information (NCBI) Sequence Read Archive under the accession number PRJNA859634, PRJNA905648, BankIt2706885, BankIt2706893, and BankIt2706894, respectively.

## Abbreviations

CFU: Colony-forming unit
UA: Urate/serum uric acid
PNP: Purine nucleoside phosphorylase
XOD: Xanthine oxidase
Cr: Creatinine
Bun: Urea nitrogen
OTU: Operational taxonomic unit
SCFA: Short-chain fatty acids
CFE: Cell-free extract
CFS: Cell-free supernatant
*P*_app_: Apparent permeability coefficient
TEER: Trans-epithelial electrical resistance
AKP: Alkaline phosphatase
