# Supplementary material for: Lactiplantibacillus plantarum enables blood urate control in mice through degradation of nucleosides in gastrointestinal tract
Source: Microbiome. 2023 Jul 19;11:153. doi: 10.1186/s40168-023-01605-y (PMC10354915; doi:10.1186/s40168-023-01605-y)
Supplement: Supplementary file 2 — Additional file 1: Table S1. Primer sequence of genes in this work. Table S2. Fifteen differential genera in mice supplemented L. plantarum when compared with HNS mice. Table S3. Taxonomy of six microbes using NCBI Nucleotide BLAST analysis tool. Table S4. Three genes involved with nucleoside degradation in L. plantarum. Table S5. Annotated genes contributed to the nucleotide and nucleoside metabolism, which showed that no enzyme in L. plantarum enable to catalyze the transformation of guanine and hypoxanthine into xanthine and resultant urate. Table S6. Genes involved with purine permeation in L. plantarum. Table S7. Permeation for nucleobases in L. plantarum within 2 hours. Table S8. Solubility of nucleosides and nucleobases in water. Table S9. Nodes information in multi-omic network analysis given by Fig. 2A. Table S10. Edges information in multi-omic network analysis given by Fig. 2A. Table S11. Nodes information in multi-omic network analysis given by Fig. S3C. Table S12. Edges information in multi-omic network analysis given by Fig. 3C. [file 40168_2023_1605_MOESM1_ESM.pdf]

Supplementary Tables

Table S1. Primer sequence of genes in this work.

Target gene	Forward (5'→3')	Reverse (5'→3')	Note
β actin	GTGCTATGTTGCTCTAGACTTCG	ATGCCACAGGATTCCATACC	Reference
SLC22A12	CCGCTTCCGACAACCTCA	CTTCTGCGCCCAAACCTATC	Urate transporter in kidney
SLC2A9	TGGACTCAATGCGATCTGG	AGAGAAGATAGCAGCCAGTGTTT	Urate transporter in kidney
SLC17A1	TGACACTTGCCAACTCAACACT	CCAAACATTCCAATTAAAGCTG	Urate transporter in kidney
ABCG2	TAGGACGCTCGCAGAAGGA	AGAATAGCATTAAGGCCAGGTTT	Urate transporter in kidney and intestine
XOD	TGATGGTTCGGTGCTGTTGA	GGGACGGTGTTAGTGCTTGT	Involved with urate synthesis in liver
PNP	GCCTGGAAACAAATGGGG	ACCAAAGACACGGAGCCC	Involved with urate synthesis in liver
ENT1	TATCGCCATTACCTGCAGCT	GACTGGTTTCTGTTGGTGGGT	Potential purine transporters in intestine
ENT2	TCTTCACGCTCCTCAACTCC	AGTCCACCTTCACCAACGC	Potential purine transporters in intestine
ENT3	CTCTCCTTGCTCTGTAACTACCA	TGCTGAGGTAGCCGTTGC	Potential purine transporters in intestine
ENT4	AGGTGACGCACAGCAACAGT	GAAGTAGGTTACCGCAATGGAC	Potential purine transporters in intestine
CNT1	GAGCCAGGATTCGTTGCG	CAAAGACATCCTTGACCAGAGC	Potential purine transporters in intestine
CNT2	AGCCAACCTGATTGCCTTTC	AGGAGCAGATGACCTGGAAACT	Potential purine transporters in intestine
CNT3	GGTCGGGTGGCTAATGCT	GACCAGTAGTGGAACTCTGTTTG	Potential purine transporters in intestine
18S	AGAATCCACGCCAGTACAAGA	TCTTCAGTCGCTCCAGGTCT	Reference
ABCG2	CTCAGTTTATCCGTGGTGTGTCT	CGTCCCTGCTTAGACATCCTT	Urate transporter in Caco-2 cells
CNT1	AGCACATGCAGCTGTTTCG	GGAAATCCAGGAGGCAGG	Potential purine transporters in Caco-2 cells
CNT2	ACTTTCCAGGTCATCTGCTCC	TTATGAAGAACTTGATTCCCACC	Potential purine transporters in Caco-2 cells
CNT3	TGCTGGTGCTTCATTTGTCTT	ACATCACAGTGCTGAAGAAAACC	Potential purine transporters in Caco-2 cells
ENT1	CCTTGCCTGAGCGGAACT	GGAAGGAGTTGAGGTAGGTGAA	Potential purine transporters in Caco-2 cells
ENT2	AGGATGCCTTCAACTTCAACAA	GAAGGAGTTGAGGAGGGTGAAG	Potential purine transporters in Caco-2 cells
ENT3	GACTCCCTCAGTGCCCCTT	ATGTTGGTGCAGATGGCG	Potential purine transporters in Caco-2 cells
ENT4	TCTTCATCCTGTGCGTCTACC	GAAGTAGCCGTTGCTGATGC	Potential purine transporters in Caco-2 cells
RihA	cagcaaatgggtcgcggatccTTGGAA GATATTATTTTAGATTGT	ctcgagtgcggccgcaagcttTTAAAAGTT G AAGGATTTGATGCT	For recombination between RihA and plasmid pET-28a
RihB	cagcaaatgggtcgcggatccATGAATG CAACCCGTAACGTTA	ctcgagtgcggccgcaagcttTTATTTTTC GCATGGCGTCA	For recombination between RihB and plasmid pET-28a
RihC	cagcaaatgggtcgcggatccATGGCAA AACGCAAAATGATT	ctcgagtgcggccgcaagcttCTAGTGTT GTTTGAACAGCGTCG	For recombination between RihC and plasmid pET-28a
RihA	ATTGGCCGTAACCACCTCAG	CGACCGAATGAGATGCCGTA	Nucleosides-degradation enzyme in L. plantarum
RihB	CTACAGCGTGACCCACAACT	GCCCCGAATACAGTCGTCAA	Nucleosides-degradation enzyme in L. plantarum
RihC	TCGAACTACCCGCACCAAAA	GTAGTCTTCACCGCTGGCTT	Nucleosides-degradation enzyme in L. plantarum

Table S2. Fifteen differential genera in mice supplemented *L. plantarum* when compared with HNS mice.

	Freq1(HNS)	Freq2(HNS+LP)	p-Value	q-Value	Difference between means	95% lower CI	95% upper CI
**g__Coriobacteriaceae**	0.3018	0.1994	0.0004	0.0295	0.1024	0.0506	0.1541
**g__Clostridium_XlVa**	5.0404	2.5219	0.0015	0.0595	2.5185	1.0814	3.9556
**g__Bacteroides**	5.0755	8.3594	0.0048	0.1228	(3.2838)	(5.4616)	(1.1060)
**g__Roseburia**	1.0102	0.3177	0.0093	0.1786	0.6925	0.1927	1.1923
**g__Enterorhabdus**	0.0609	0.0379	0.0171	0.2637	0.0229	0.0044	0.0415
**g__Alphaproteobacteria**	0.0032	0.0002	0.0191	0.2446	0.0030	0.0006	0.0054
**g__Lachnospiraceae**	27.0497	30.5853	0.0259	0.2853	(3.5356)	(6.6206)	(0.4507)
**g__Parvibacter**	0.0417	0.0151	0.0323	0.3113	0.0266	0.0025	0.0506
**g__Oscillibacter**	1.7768	2.4179	0.0340	0.2910	(0.6411)	(1.2307)	(0.0515)
**g__Mucispirillum**	0.0313	0.0580	0.0359	0.2766	(0.0267)	(0.0515)	(0.0019)
**g__Holdemania**	0.0013	0.0044	0.0360	0.2518	(0.0032)	(0.0062)	(0.0002)
**g__Bacteroidetes**	0.0225	0.0991	0.0415	0.2664	(0.0766)	(0.1499)	(0.0033)
**g__Flavonifractor**	0.5902	0.9367	0.0447	0.2647	(0.3465)	(0.6843)	(0.0087)
**g__Staphylococcus**	0.0001	0.0011	0.0455	0.2504	(0.0010)	(0.0019)	(0.0000)
**g__Rhodospirillaceae**	0.0259	0.0151	0.0488	0.2506	0.0108	0.0001	0.0216

Table S3. Taxonomy of six microbes using NCBI Nucleotide BLAST analysis tool. https://blast.ncbi.nlm.nih.gov/Blast.cgi?PROGRAM=blastn&PAGE_TYPE=BlastSearch&LINK_LOC=blasthome

OUT	BLAST from NCBI
OTU26	Lactobacillus johnsonii
OTU31	Limosilactobacillus reuteri
OTU28	Ligilactobacillus murinus
OTU130	Lactobacillus intestinalis
OTU38	Uncultured bacterium
OTU356	Uncultured bacterium

Table S4. Three genes involved with nucleoside degradation in *L. plantarum*.

Sequence ID	COG	KEGG	Predictor description	Name
**PROKKA_00051**	6e-110 gnl|CDD|224868 COG1957, URH1, Inosine-uridine nucleoside N-ribohydrolase [Nucleotide transport and metabolism].	K01239-ko00230,Purine metabolism;ko00760,Nicotinate and nicotinamide metabolism	Pyrimidine-specific ribonucleoside hydrolase	RihA
**PROKKA_01337**	2e-96 gnl|CDD|224868 COG1957, URH1, Inosine-uridine nucleoside N-ribohydrolase [Nucleotide transport and metabolism].	K01239-ko00230,Purine metabolism;ko00760,Nicotinate and nicotinamide metabolism	Pyrimidine-specific ribonucleoside hydrolase	RihB
**PROKKA_00511**	8e-105 gnl|CDD|224868 COG1957, URH1, Inosine-uridine nucleoside N-ribohydrolase [Nucleotide transport and metabolism].	K01239-ko00230,Purine metabolism;ko00760,Nicotinate and nicotinamide metabolism	Non-specific ribonucleoside hydrolase	RihC

Table S5. Annotated genes contributed to the nucleotide and nucleoside metabolism, which showed that no enzyme in *L. plantarum* enable to catalyze the transformation of guanine and hypoxanthine into xanthine and resultant urate.

Inosine-uridine nucleoside N-ribohydrolase; Aspartate carbamoyltransferase, catalytic chain; ADP-ribose pyrophosphatase; GMP synthase - Glutamine amidotransferase domain; Thymidylate kinase; Purine-cytosine permease and related proteins; ADP-ribose pyrophosphatase; Nucleoside 2-deoxyribosyltransferase; Nucleoside diphosphate kinase; Inosine-uridine nucleoside N-ribohydrolase; Phosphoribosylaminoimidazole carboxylase (NCAIR synthetase); Adenine/guanine phosphoribosyltransferases and related PRPP-binding proteins; CTP synthase (UTP-ammonia lyase); Deoxyribose-phosphate aldolase; Carbamoylphosphate synthase large subunit (split gene in MJ); Carbamoylphosphate synthase small subunit; Hypoxanthine-guanine phosphoribosyltransferase; Inosine-uridine nucleoside N-ribohydrolase; Deoxycytidylate deaminase; Adenine/guanine phosphoribosyltransferases and related PRPP-binding proteins; ADP-ribose pyrophosphatase; Nucleoside phosphorylaseDihydroorotate dehydrogenase; Carbamoylphosphate synthase large subunit (split gene in MJ); Carbamoylphosphate synthase small subunit; Dihydroorotase and related cyclic amidohydrolases; Aspartate carbamoyltransferase, catalytic chain; Pyrimidine operon attenuation protein/uracil phosphoribosyltransferase; Xanthine/uracil permeases; Phosphoribosylamine-glycine ligase; AICAR transformylase/IMP cyclohydrolase PurH (only IMP cyclohydrolase domain in Aful); Folate-dependent phosphoribosylglycinamide formyltransferase PurN; Phosphoribosylaminoimidazole (AIR) synthetase; Glutamine phosphoribosylpyrophosphate amidotransferase; Phosphoribosylformylglycinamidine (FGAM) synthase, synthetase domain; Phosphoribosylformylglycinamidine (FGAM) synthase, glutamine amidotransferase domain; Phosphoribosylformylglycinamidine (FGAM) synthase, PurS component; Phosphoribosylaminoimidazolesuccinocarboxamide (SAICAR) synthase; Phosphoribosylcarboxyaminoimidazole (NCAIR) mutase; dUTPase; 5'-nucleotidase/2',3'-cyclic phosphodiesterase and related esterases; Xanthosine triphosphate pyrophosphatase; Inosine-uridine nucleoside N-ribohydrolase; Phosphoribosylaminoimidazole carboxylase (NCAIR synthetase); Adenine/guanine phosphoribosyltransferases and related PRPP-binding proteins; Adenylate kinase and related kinases; Deoxynucleoside kinases; Uridine kinase; GMP synthase, PP-ATPase domain/subunit; ADP-ribose pyrophosphatase; Guanylate kinase; ADP-ribose pyrophosphatase; Thymidylate kinase; Ribonucleotide reductase, beta subunit; Ribonucleotide reductase, alpha subunit; Protein involved in ribonucleotide reduction; Oxygen-sensitive ribonucleoside-triphosphate reductase; IMP dehydrogenase/GMP reductase; Nucleoside permease; Adenylosuccinate lyase; Adenylosuccinate synthase; IMP dehydrogenase/GMP reductase; Xanthosine triphosphate pyrophosphatase; ADP-ribose pyrophosphatase; Uridine kinase; Guanylate kinase; Pyrimidine operon attenuation protein/uracil phosphoribosyltransferase; Carbamoylphosphate synthase small subunit; Carbamoylphosphate synthase large subunit (split gene in MJ); Thymidylate synthase; Cytidylate kinase; ADP-ribose pyrophosphatase; Thymidine kinase; Uracil phosphoribosyltransferase; Xanthine/uracil permeases; Uridine phosphorylase; 5'-nucleotidase/2',3'-cyclic phosphodiesterase and related esterases; Nucleoside 2-deoxyribosyltransferase; Purine-cytosine permease and related proteins; Adenine deaminase; Deoxynucleoside kinases; Adenine/guanine phosphoribosyltransferases and related PRPP-binding proteins; Formyltetrahydrofolate synthetase

Table S6. Genes involved with purine permeation in *L. plantarum*.

Gene ID	Predictor description	Function in Lactobacillus
**PROKKA_01052**	Uric acid permease PucK	https://www.orthodb.org/?level=&species=&query=5237at1578
**PROKKA_01053**	Guanine/hypoxanthine permease PbuG	https://www.orthodb.org/?level=&species=&query=5582at1578
**PROKKA_01673**	Guanine/hypoxanthine permease PbuG	https://www.orthodb.org/?level=&species=&query=5582at1578

Table S7. Permeation for nucleobases in *L. plantarum* within 2 hours.

Content in cell %	0h			1h			2h			p-Value 0h vs 1h	p-Value 1h vs 2h	p-Value 0h vs 2h
	1	2	3	1	2	3	1	2	3			
Hypoxanthine	0.2	0.7	0.5	1.7	1.8	1.6	3.5	3.4	3.7	0.00952	0.00581	0.00332
Guanine	0.2	0.3	0.3	1.1	0.9	1.1	3.1	2.8	3.1	0.01065	0.00020	0.00153

Table S8. Solubility of nucleosides and nucleobases in water

Substance	ChemSpider ID	Water Solubility Estimate from Log Kow (WSKOW v1.41) mg/L, 25 ºC	Water Sol Estimate from Fragments (v1.01 est) mg/L	Water Solubility in Chemical Book g/L
Inosine	21241953	1.00E+06	1.00E+06	21
Guanosine	6544	1.00E+06	1.00E+06	0.75
Hypoxanthine	11205977	1.26E+04	1874.9	Practically insoluble
Guanine	744	1.00E+06	1.00E+06	Practically insoluble

Table S9. Nodes information in multi-omic network analysis given by Fig. 2A.

Group	Node	Weight degree	Eigenvector centrality	Betweenness centrality	Closeness centrality	Degree
gene	GLUT9	7.3287	6.16E-04	174	2.47E-04	6
gene	NPT1	5.8494	2.15E-01	228	4.61E-04	9
gene	XOD	1.4052	1.02E-02	0	4.50E-04	2
gene	PNP	4.3481	1.02E-01	189	4.60E-04	7
gene	URAT1	7.2403	1.72E-01	80	4.60E-04	11
gene	ABCG2	2.9662	3.91E-02	0	4.52E-04	4
metabolite	Serum UA	3.6442	3.04E-02	24	4.53E-04	5
microbiota	OTU7	3.1364	8.88E-02	31	4.59E-04	5
microbiota	OTU73	0.6403	2.17E-04	0	4.36E-04	1
microbiota	OTU82	0.4159	2.06E-04	0	4.76E-04	1
microbiota	OTU1	0.3479	2.46E-02	0	4.21E-04	1
microbiota	OTU2	7.7468	6.40E-01	76.5	4.64E-04	12
microbiota	OTU1	12.8182	5.01E-01	196	4.65E-04	20
microbiota	OTU6	9.5013	2.91E-01	178.5	4.63E-04	15
microbiota	OTU14	4.7805	9.03E-02	55	4.59E-04	8
microbiota	OTU13	9.2961	6.11E-01	358	4.65E-04	15
microbiota	OTU24	6.7636	1.35E-01	63	4.59E-04	10
microbiota	OTU63	7.5299	1.24E-01	226	4.60E-04	12
microbiota	OTU378	6.8948	1.51E-01	60	4.60E-04	11
microbiota	OTU33	6.3286	1.27E-01	77	4.60E-04	10
microbiota	OTU67	4.1727	2.19E-01	63	4.61E-04	7
microbiota	OTU321	7.2675	1.64E-01	24	4.60E-04	11
microbiota	OTU36	6.9610	1.60E-01	67	4.60E-04	11
microbiota	OTU70	11.1429	8.84E-01	60	4.63E-04	17
microbiota	OTU39	9.7883	2.16E-01	578	4.64E-04	16
microbiota	OTU40	10.7870	9.00E-01	15	4.63E-04	16
microbiota	OTU71	6.0532	4.01E-01	92	4.63E-04	10
microbiota	OTU55	9.2195	6.44E-01	169.5	4.65E-04	15
microbiota	OTU52	4.4468	2.81E-01	8	4.60E-04	7
microbiota	OTU65	4.2740	2.25E-01	95.5	4.61E-04	7
microbiota	OTU15	9.7870	7.31E-01	92	4.62E-04	15
microbiota	OTU10	9.9519	8.17E-01	160	4.62E-04	15
microbiota	OTU59	9.9416	6.68E-01	282	4.63E-04	16
microbiota	OTU42	10.0078	7.52E-01	76	4.63E-04	16
microbiota	OTU142	13.2494	9.18E-01	290.5	4.65E-04	21
microbiota	OTU44	9.4221	6.22E-01	288	4.63E-04	15
microbiota	OTU60	13.3403	1.00E+00	159.25	4.64E-04	21
microbiota	OTU107	1.9286	8.76E-02	0	4.58E-04	3
microbiota	OTU57	2.5584	6.06E-02	0	4.55E-04	4
microbiota	OTU49	3.0545	6.73E-02	61	4.58E-04	5
microbiota	OTU34	1.2688	1.42E-02	0	4.52E-04	2
microbiota	OTU111	5.6130	7.91E-02	107	4.60E-04	9
microbiota	OTU16	8.0208	4.81E-01	279	4.64E-04	13
microbiota	OTU83	5.6078	3.58E-01	50	4.62E-04	9
microbiota	OTU104	9.3766	7.53E-01	68	4.62E-04	15
microbiota	OTU62	3.3299	3.03E-02	22	4.54E-04	5
microbiota	OTU47	1.8455	1.27E-02	4	4.50E-04	3

microbiota	OTU66	1.2935	1.35E-02	0	4.50E-04	2
microbiota	OTU106	2.4481	3.83E-02	79	4.55E-04	4
microbiota	OTU356	2.4403	1.58E-02	235	4.55E-04	4
microbiota	OTU8	13.8065	9.71E-01	198.5	4.64E-04	21
microbiota	OTU25	3.6974	2.28E-01	55	4.59E-04	6
microbiota	OTU56	10.8221	8.37E-01	77.75	4.63E-04	16
microbiota	OTU292	4.4312	3.16E-01	118	4.60E-04	7
microbiota	OTU32	2.9532	2.43E-01	4	4.58E-04	5
microbiota	OTU76	2.5104	2.32E-01	0	4.56E-04	4
microbiota	OTU295	3.5078	1.75E-01	25	4.56E-04	5
microbiota	OTU336	5.5325	3.38E-01	170	4.60E-04	9
microbiota	OTU51	4.1870	1.88E-01	99	4.60E-04	7
microbiota	OTU53	1.7299	3.60E-02	87	4.52E-04	3
microbiota	OTU93	3.6896	3.35E-01	2	4.58E-04	6
microbiota	OTU109	3.6532	2.79E-01	6	4.58E-04	6
microbiota	OTU64	4.8364	2.44E-01	206	4.60E-04	8
microbiota	OTU61	3.6091	2.49E-01	11	4.59E-04	6
microbiota	OTU37	1.1325	4.66E-02	0	4.58E-04	2
microbiota	OTU127	1.2701	4.08E-02	0	4.55E-04	2
microbiota	OTU43	3.8649	7.41E-02	199	4.55E-04	6
microbiota	OTU45	5.6273	2.37E-01	58	4.60E-04	9
microbiota	OTU141	0.6455	4.77E-02	0	4.51E-04	1
microbiota	OTU5	9.0636	6.55E-01	83	4.61E-04	14
microbiota	OTU19	5.0299	9.54E-02	44	4.59E-04	8
microbiota	OTU9	2.5000	3.55E-02	11	4.55E-04	4
microbiota	OTU21	1.7688	1.15E-02	21	4.50E-04	3
microbiota	OTU4	1.2429	1.86E-02	0	4.47E-04	2
microbiota	OTU48	1.2896	7.67E-03	12	4.47E-04	2
microbiota	OTU58	0.5935	5.11E-03	0	4.45E-04	1
microbiota	OTU20	1.9481	7.72E-02	2	4.55E-04	3
microbiota	OTU221	1.9000	7.28E-02	0	4.55E-04	3
microbiota	OTU17	3.5429	1.01E-01	55	4.58E-04	6
microbiota	OTU30	2.5857	1.33E-01	228	4.58E-04	4
microbiota	OTU46	1.2961	9.22E-03	86	4.47E-04	2
microbiota	OTU22	1.1701	1.61E-02	86	4.50E-04	2
microbiota	OTU29	0.5870	5.89E-04	0	4.40E-04	1
microbiota	OTU11	0.5545	1.93E-03	0	4.42E-04	1
microbiota	OTU38	0.6701	0.00E+00	0	4.32E-04	1
microbiota	OTU31	2.0390	1.01E-03	93	4.43E-04	3
microbiota	OTU26	3.2571	2.34E-02	115	4.54E-04	5
microbiota	OTU130	1.2506	6.45E-04	60	4.45E-04	2
microbiota	OTU35	0.5494	4.05E-02	0	4.53E-04	1
microbiota	OTU41	1.7156	2.49E-02	59	4.53E-04	3
microbiota	OTU28	0.5857	7.59E-04	0	4.45E-04	1

Table S10. Edges information in multi-omic network analysis given by Fig. 2A.

Node 1	Node 2	Correlation	Relationship	Weight
Serum UA	ABCG2	-0.8247	negative	0.82
OTU26	ABCG2	-0.7636	negative	0.76
OTU73	GLUT9	-0.8252	negative	0.83
OTU82	GLUT9	-0.7832	negative	0.78
URAT1	GLUT9	-0.7124	negative	0.71
NPT1	GLUT9	0.7152	positive	0.72
OTU7	GLUT9	0.7203	positive	0.72
Serum UA	GLUT9	0.8958	positive	0.90
OTU56	NPT1	-0.7506	negative	0.75
Serum UA	NPT1	-0.7130	negative	0.71
OTU26	NPT1	-0.7253	negative	0.73
OTU10	NPT1	-0.7058	negative	0.71
OTU49	NPT1	0.7417	positive	0.74
OTU1	NPT1	0.7494	positive	0.75
OTU71	NPT1	0.7039	positive	0.70
ABCG2	NPT1	0.8208	positive	0.82
OTU15	OTU10	-0.7870	negative	0.79
OTU5	OTU10	-0.7766	negative	0.78
OTU2	OTU10	-0.7097	negative	0.71
OTU8	OTU10	0.7208	positive	0.72
OTU56	OTU104	-0.7987	negative	0.80
OTU10	OTU104	-0.7688	negative	0.77
OTU8	OTU104	-0.7519	negative	0.75
OTU142	OTU104	-0.7487	negative	0.75
OTU13	OTU104	-0.7123	negative	0.71
OTU61	OTU104	0.7006	positive	0.70
OTU70	OTU104	0.7084	positive	0.71
OTU32	OTU104	0.7136	positive	0.71
OTU15	OTU104	0.7240	positive	0.72
OTU83	OTU104	0.7326	positive	0.73
OTU45	OTU104	0.7443	positive	0.74
OTU40	OTU104	0.7286	positive	0.73
OTU44	OTU104	0.7119	positive	0.71
OTU5	OTU104	0.7104	positive	0.71
OTU66	OTU106	0.7019	positive	0.70
OTU41	OTU106	0.7118	positive	0.71
OTU63	OTU106	0.7325	positive	0.73
OTU336	OTU106	0.7218	positive	0.72
OTU13	OTU107	-0.7727	negative	0.77
OTU6	OTU107	0.7314	positive	0.73
OTU10	OTU109	-0.7448	negative	0.74
OTU93	OTU109	0.7266	positive	0.73
OTU5	OTU109	0.7300	positive	0.73
OTU336	OTU109	0.7286	positive	0.73
OTU15	OTU109	0.7494	positive	0.75
OTU51	OTU111	0.7019	positive	0.70
OTU14	OTU111	0.7110	positive	0.71

OTU62	OTU111	0.7222	positive	0.72
OTU36	OTU111	0.7352	positive	0.74
OTU39	OTU111	0.7352	positive	0.74
OTU127	OTU111	0.7236	positive	0.72
OTU24	OTU111	0.7296	positive	0.73
OTU33	OTU111	0.7260	positive	0.73
OTU16	OTU127	0.7365	positive	0.74
OTU6	OTU13	-0.7156	negative	0.72
OTU1	OTU13	-0.7214	negative	0.72
OTU30	OTU13	0.7469	positive	0.75
OTU8	OTU13	0.7140	positive	0.71
OTU31	OTU130	0.7157	positive	0.72
OTU356	OTU130	0.7249	positive	0.72
OTU1	OTU14	0.7019	positive	0.70
OTU19	OTU14	0.7339	positive	0.73
OTU44	OTU141	0.7455	positive	0.75
OTU292	OTU142	-0.7312	negative	0.73
OTU59	OTU142	-0.7338	negative	0.73
OTU16	OTU142	-0.7065	negative	0.71
OTU2	OTU142	-0.7039	negative	0.70
OTU25	OTU142	-0.7396	negative	0.74
OTU42	OTU142	-0.7279	negative	0.73
OTU64	OTU142	-0.7123	negative	0.71
OTU56	OTU142	0.7110	positive	0.71
OTU10	OTU142	0.7352	positive	0.74
OTU13	OTU142	0.7078	positive	0.71
OTU30	OTU142	0.7610	positive	0.76
OTU8	OTU142	0.8143	positive	0.81
OTU2	OTU15	0.7171	positive	0.72
OTU13	OTU16	-0.7104	negative	0.71
OTU8	OTU16	-0.7234	negative	0.72
OTU292	OTU16	0.7175	positive	0.72
OTU7	OTU16	0.7094	positive	0.71
OTU221	OTU17	0.7430	positive	0.74
OTU1	OTU2	0.7296	positive	0.73
OTU5	OTU20	0.7106	positive	0.71
OTU9	OTU21	-0.7143	negative	0.71
OTU6	OTU24	0.7313	positive	0.73
OTU1	OTU24	0.7335	positive	0.73
OTU8	OTU25	-0.7519	negative	0.75
OTU31	OTU26	0.7831	positive	0.78
OTU22	OTU29	-0.7370	negative	0.74
OTU8	OTU292	-0.7156	negative	0.72
OTU142	OTU295	-0.7740	negative	0.77
OTU8	OTU295	-0.7110	negative	0.71
OTU4	OTU295	0.7283	positive	0.73
OTU292	OTU295	0.7636	positive	0.76
OTU43	OTU295	0.8208	positive	0.82
OTU38	OTU31	0.7301	positive	0.73
OTU8	OTU32	-0.7084	negative	0.71

OTU59	OTU32	0.7149	positive	0.71
OTU24	OTU321	-0.7026	negative	0.70
OTU1	OTU321	-0.7870	negative	0.79
OTU33	OTU321	-0.7753	negative	0.78
OTU14	OTU321	-0.7104	negative	0.71
OTU19	OTU321	-0.7026	negative	0.70
OTU6	OTU321	-0.7318	negative	0.73
OTU63	OTU321	0.7032	positive	0.70
OTU378	OTU321	0.7403	positive	0.74
OTU63	OTU33	-0.7253	negative	0.73
OTU378	OTU33	-0.7214	negative	0.72
OTU7	OTU33	0.7019	positive	0.70
OTU14	OTU33	0.7071	positive	0.71
OTU1	OTU33	0.7105	positive	0.71
OTU24	OTU33	0.7117	positive	0.71
OTU10	OTU336	-0.7039	negative	0.70
OTU8	OTU336	-0.7097	negative	0.71
OTU142	OTU336	-0.7032	negative	0.70
OTU15	OTU336	0.7006	positive	0.70
OTU41	OTU336	0.7227	positive	0.72
OTU25	OTU336	0.7442	positive	0.74
OTU42	OTU336	0.7377	positive	0.74
OTU14	OTU34	-0.7481	negative	0.75
OTU39	OTU356	0.7149	positive	0.71
OTU28	OTU356	0.7157	positive	0.72
OTU62	OTU356	0.7547	positive	0.75
OTU321	OTU36	-0.7545	negative	0.75
OTU6	OTU36	0.7045	positive	0.70
OTU51	OTU36	0.7240	positive	0.72
OTU14	OTU36	0.7365	positive	0.74
OTU20	OTU36	0.7378	positive	0.74
OTU1	OTU36	0.7312	positive	0.73
OTU24	OTU36	0.7338	positive	0.73
OTU33	OTU36	0.8169	positive	0.82
OTU16	OTU37	0.7006	positive	0.70
OTU1	OTU378	-0.7844	negative	0.78
OTU19	OTU378	-0.7273	negative	0.73
OTU9	OTU378	-0.7474	negative	0.75
OTU6	OTU378	-0.7162	negative	0.72
OTU63	OTU378	0.7275	positive	0.73
OTU378	OTU39	-0.7182	negative	0.72
OTU63	OTU39	-0.7409	negative	0.74
OTU13	OTU39	-0.7110	negative	0.71
OTU33	OTU39	0.7123	positive	0.71
OTU16	OTU39	0.7279	positive	0.73
OTU37	OTU39	0.7118	positive	0.71
OTU107	OTU39	0.7144	positive	0.71
OTU36	OTU39	0.7430	positive	0.74
OTU1	OTU39	0.7495	positive	0.75
OTU9	OTU39	0.7442	positive	0.74

OTU6	OTU39	0.7922	positive	0.79
OTU10	OTU40	-0.7468	negative	0.75
OTU56	OTU40	-0.7234	negative	0.72
OTU8	OTU40	-0.7416	negative	0.74
OTU142	OTU40	-0.7435	negative	0.74
OTU32	OTU40	0.7521	positive	0.75
OTU1	OTU40	0.7106	positive	0.71
OTU44	OTU40	0.7314	positive	0.73
OTU42	OTU40	0.7169	positive	0.72
OTU2	OTU40	0.7338	positive	0.73
OTU5	OTU40	0.7455	positive	0.75
OTU15	OTU40	0.7610	positive	0.76
OTU70	OTU40	0.7766	positive	0.78
OTU8	OTU42	-0.7052	negative	0.71
OTU13	OTU42	-0.7006	negative	0.70
OTU5	OTU42	0.7274	positive	0.73
OTU15	OTU42	0.7456	positive	0.75
OTU2	OTU42	0.7416	positive	0.74
OTU59	OTU42	0.7210	positive	0.72
OTU16	OTU42	0.7065	positive	0.71
OTU30	OTU43	-0.7019	negative	0.70
OTU4	OTU43	0.7045	positive	0.70
OTU59	OTU43	0.7456	positive	0.75
OTU56	OTU44	-0.7091	negative	0.71
OTU142	OTU44	-0.7273	negative	0.73
OTU17	OTU44	-0.7006	negative	0.70
OTU8	OTU44	-0.7006	negative	0.70
OTU35	OTU44	-0.6994	negative	0.70
OTU5	OTU44	0.7170	positive	0.72
OTU59	OTU44	0.7261	positive	0.73
OTU15	OTU44	0.7106	positive	0.71
OTU2	OTU44	0.7156	positive	0.72
OTU17	OTU45	-0.7279	negative	0.73
OTU64	OTU45	0.7279	positive	0.73
OTU5	OTU45	0.7404	positive	0.74
OTU19	OTU45	0.7299	positive	0.73
OTU7	OTU45	0.7390	positive	0.74
OTU32	OTU45	0.7442	positive	0.74
OTU59	OTU45	0.7468	positive	0.75
OTU73	OTU46	0.7403	positive	0.74
OTU30	OTU46	0.7158	positive	0.72
OTU21	OTU47	-0.7123	negative	0.71
OTU62	OTU47	0.7312	positive	0.73
OTU24	OTU47	0.7119	positive	0.71
OTU41	OTU48	0.7110	positive	0.71
OTU43	OTU48	0.7286	positive	0.73
OTU26	OTU49	-0.7214	negative	0.72
OTU39	OTU49	0.7222	positive	0.72
OTU6	OTU49	0.7235	positive	0.72
OTU15	OTU5	0.8597	positive	0.86

OTU25	OTU51	0.7097	positive	0.71
OTU16	OTU51	0.7201	positive	0.72
OTU15	OTU51	0.7157	positive	0.72
OTU20	OTU51	0.7296	positive	0.73
OTU42	OTU52	0.7006	positive	0.70
OTU67	OTU52	0.7253	positive	0.73
OTU6	OTU52	0.7403	positive	0.74
OTU40	OTU52	0.7094	positive	0.71
OTU36	OTU52	0.7236	positive	0.72
OTU1	OTU52	0.7166	positive	0.72
OTU70	OTU52	0.7309	positive	0.73
OTU292	OTU53	0.7006	positive	0.70
OTU11	OTU53	0.7045	positive	0.70
OTU25	OTU53	0.7547	positive	0.75
OTU8	OTU55	-0.8130	negative	0.81
OTU142	OTU55	-0.6987	negative	0.70
OTU13	OTU55	-0.7214	negative	0.72
OTU42	OTU55	0.7084	positive	0.71
OTU40	OTU55	0.7110	positive	0.71
OTU71	OTU55	0.7149	positive	0.71
OTU1	OTU55	0.7144	positive	0.71
OTU16	OTU55	0.7365	positive	0.74
OTU59	OTU55	0.7508	positive	0.75
OTU25	OTU55	0.7273	positive	0.73
OTU292	OTU55	0.7455	positive	0.75
OTU44	OTU55	0.7166	positive	0.72
OTU5	OTU56	-0.7013	negative	0.70
OTU15	OTU56	-0.7831	negative	0.78
OTU93	OTU56	-0.7143	negative	0.71
OTU8	OTU56	0.7288	positive	0.73
OTU10	OTU56	0.8013	positive	0.80
OTU63	OTU57	-0.7675	negative	0.77
OTU378	OTU57	-0.7253	negative	0.73
OTU6	OTU57	0.7469	positive	0.75
OTU39	OTU57	0.7387	positive	0.74
OTU43	OTU58	-0.7435	negative	0.74
OTU56	OTU59	-0.7831	negative	0.78
OTU8	OTU59	-0.7442	negative	0.74
OTU10	OTU59	-0.7214	negative	0.72
OTU221	OTU59	-0.7162	negative	0.72
OTU2	OTU59	0.7339	positive	0.73
OTU16	OTU59	0.7119	positive	0.71
OTU2	OTU6	0.7196	positive	0.72
OTU1	OTU6	0.7675	positive	0.77
OTU59	OTU60	-0.7312	negative	0.73
OTU70	OTU60	-0.7091	negative	0.71
OTU93	OTU60	-0.7052	negative	0.71
OTU104	OTU60	-0.7416	negative	0.74
OTU71	OTU60	-0.7130	negative	0.71
OTU109	OTU60	-0.7039	negative	0.70

OTU16	OTU60	-0.7435	negative	0.74
OTU61	OTU60	-0.7396	negative	0.74
OTU15	OTU60	-0.7344	negative	0.73
OTU42	OTU60	-0.7188	negative	0.72
OTU2	OTU60	-0.7149	negative	0.71
OTU40	OTU60	-0.7149	negative	0.71
OTU55	OTU60	-0.7110	negative	0.71
OTU292	OTU60	-0.7071	negative	0.71
OTU5	OTU60	-0.7058	negative	0.71
OTU13	OTU60	0.7136	positive	0.71
OTU76	OTU60	0.7326	positive	0.73
OTU142	OTU60	0.7547	positive	0.75
OTU10	OTU60	0.7935	positive	0.79
OTU8	OTU60	0.8052	positive	0.81
OTU56	OTU60	0.8065	positive	0.81
OTU56	OTU61	-0.7377	negative	0.74
OTU17	OTU61	-0.7143	negative	0.71
OTU5	OTU61	0.6994	positive	0.70
OTU24	OTU62	0.8208	positive	0.82
OTU24	OTU63	-0.7714	negative	0.77
OTU62	OTU63	-0.7610	negative	0.76
OTU1	OTU63	-0.7182	negative	0.72
OTU6	OTU63	-0.7084	negative	0.71
OTU21	OTU63	0.7222	positive	0.72
OTU221	OTU64	-0.7208	negative	0.72
OTU56	OTU64	-0.7344	negative	0.73
OTU15	OTU64	0.7131	positive	0.71
OTU22	OTU64	0.7131	positive	0.71
OTU5	OTU64	0.7196	positive	0.72
OTU7	OTU64	0.7351	positive	0.74
OTU321	OTU65	-0.7182	negative	0.72
OTU56	OTU65	-0.7097	negative	0.71
OTU55	OTU65	0.7071	positive	0.71
OTU24	OTU65	0.7170	positive	0.72
OTU1	OTU65	0.7443	positive	0.74
OTU61	OTU65	0.7275	positive	0.73
OTU44	OTU65	0.7301	positive	0.73
OTU63	OTU66	0.7416	positive	0.74
OTU17	OTU67	-0.7078	negative	0.71
OTU378	OTU67	-0.7240	negative	0.72
OTU59	OTU67	0.7274	positive	0.73
OTU2	OTU67	0.7300	positive	0.73
OTU42	OTU67	0.7326	positive	0.73
OTU1	OTU67	0.7456	positive	0.75
OTU19	OTU7	0.7210	positive	0.72
OTU10	OTU70	-0.7792	negative	0.78
OTU56	OTU70	-0.7390	negative	0.74
OTU8	OTU70	-0.7286	negative	0.73
OTU13	OTU70	-0.7357	negative	0.74
OTU142	OTU70	-0.7097	negative	0.71

OTU93	OTU70	0.7058	positive	0.71
OTU1	OTU70	0.7469	positive	0.75
OTU5	OTU70	0.7299	positive	0.73
OTU51	OTU70	0.7158	positive	0.72
OTU42	OTU70	0.7117	positive	0.71
OTU2	OTU70	0.7870	positive	0.79
OTU15	OTU70	0.7948	positive	0.79
OTU8	OTU71	-0.7065	negative	0.71
OTU13	OTU71	-0.7422	negative	0.74
OTU70	OTU71	0.7136	positive	0.71
OTU6	OTU71	0.7547	positive	0.75
OTU1	OTU71	0.7260	positive	0.73
OTU42	OTU76	-0.7052	negative	0.71
OTU8	OTU76	0.7000	positive	0.70
OTU8	OTU83	-0.7117	negative	0.71
OTU13	OTU83	-0.7403	negative	0.74
OTU76	OTU83	-0.7026	negative	0.70
OTU142	OTU83	-0.7149	negative	0.71
OTU59	OTU83	0.7032	positive	0.70
OTU71	OTU83	0.7352	positive	0.74
OTU19	OTU83	0.7403	positive	0.74
OTU45	OTU83	0.7270	positive	0.73
OTU19	OTU9	0.7442	positive	0.74
OTU10	OTU93	-0.7870	negative	0.79
OTU15	OTU93	0.7006	positive	0.70
OTU111	PNP	-0.7182	negative	0.72
OTU55	PNP	-0.7227	negative	0.72
OTU44	PNP	-0.7201	negative	0.72
OTU17	PNP	0.7092	positive	0.71
GLUT9	PNP	0.7276	positive	0.73
OTU34	PNP	0.7508	positive	0.75
Serum UA	PNP	0.7623	positive	0.76
OTU26	Serum UA	0.8630	positive	0.86
OTU321	URAT1	-0.8416	negative	0.84
OTU378	URAT1	-0.7727	negative	0.77
OTU71	URAT1	0.7032	positive	0.70
OTU39	URAT1	0.7118	positive	0.71
OTU19	URAT1	0.7508	positive	0.75
OTU6	URAT1	0.7273	positive	0.73
OTU14	URAT1	0.7416	positive	0.74
ABCG2	URAT1	0.7171	positive	0.72
NPT1	URAT1	0.7288	positive	0.73
OTU49	URAT1	0.7257	positive	0.73
OTU1	URAT1	0.7296	positive	0.73
PNP	XOD	0.7547	positive	0.75
GLUT9	XOD	0.7284	positive	0.73
Serum UA	XOD	0.7805	positive	0.78

Table S11. Nodes information in multi-omic network analysis given by Fig. S3C.

Group	Node	Weight degree	Eigenvector centrality	Betweenness centrality	Closeness centrality	Degree
serum metabolite	Serum UA	8.4148	9.29E-06	986	2.83E-04	10
gut metabolite	Valine	1.5294	3.94E-03	175	2.70E-04	2
gut metabolite	Taurine	2.2907	5.72E-03	652	2.76E-04	3
gut metabolite	Creatine	0.7380	3.33E-04	0	2.65E-04	1
gut metabolite	Choline	4.6064	1.31E-04	0	2.62E-04	6
gut metabolite	Alphaarabinose	2.9457	2.35E-01	0	2.75E-04	4
gut metabolite	Nicotinate	1.6054	7.85E-04	91	2.68E-04	2
gut metabolite	Tryptophan	1.5625	0.00E+00	300	2.62E-04	2
gut metabolite	Hypoxanthine	1.6766	0.00E+00	0	2.66E-04	2
gut metabolite	Urocanate	2.3853	0.00E+00	155	2.69E-04	3
gut metabolite	Butyrate	2.3814	2.36E-05	98	2.69E-04	3
gut metabolite	Betagalactose	7.0390	8.84E-03	1115	2.81E-04	9
gut metabolite	Alphaglucose	2.3303	0.00E+00	0	2.73E-04	3
gut metabolite	Lactate	6.6441	0.00E+00	735	2.74E-04	8
gut metabolite	Propionate	7.4718	0.00E+00	626	2.81E-04	9
gut metabolite	Betaxylose	7.2395	0.00E+00	1171	2.78E-04	9
gut metabolite	Isoleucine	6.7953	0.00E+00	0	2.81E-04	8
gut metabolite	Glycine	6.5973	0.00E+00	87	2.80E-04	8
gut metabolite	Phenylacetate	6.8500	0.00E+00	73	2.75E-04	8
gut metabolite	Betaglucose	3.3046	7.01E-04	860	2.79E-04	4
microbiota	OTU145	0.7888	0.00E+00	0	2.64E-04	1
microbiota	OTU124	0.8310	0.00E+00	0	2.33E-04	1
microbiota	OTU95	0.8347	0.00E+00	0	2.53E-04	1
microbiota	OTU157	1.6489	0.00E+00	206	2.42E-04	2
microbiota	OTU102	0.9150	0.00E+00	0	2.45E-04	1
microbiota	OTU125	0.7363	1.08E-04	0	2.65E-04	1
microbiota	OTU108	1.4844	5.70E-04	0	2.65E-04	2
microbiota	OTU130	2.2408	3.14E-04	247	2.77E-04	3
microbiota	OTU27	3.1110	3.98E-04	237	2.79E-04	4
microbiota	OTU114	1.4515	2.10E-05	415	2.65E-04	2
microbiota	OTU176	1.5896	0.00E+00	410	2.52E-04	2
microbiota	OTU166	1.5010	0.00E+00	0	2.69E-04	2
microbiota	OTU549	3.1361	3.29E-04	262	2.75E-04	4
microbiota	OTU160	2.5705	0.00E+00	206	2.65E-04	3
microbiota	OTU118	1.6277	6.20E-05	130	2.72E-04	2
microbiota	OTU175	1.5256	3.51E-06	76	2.63E-04	2
microbiota	OTU69	1.5964	2.77E-03	13	2.70E-04	2
microbiota	OTU435	2.9804	5.27E-03	583	2.80E-04	4
microbiota	OTU57	0.7539	0.00E+00	0	2.55E-04	1
microbiota	OTU588	3.8465	1.13E-02	2324	2.87E-04	5
microbiota	OTU235	3.7825	1.54E-03	536	2.83E-04	5
microbiota	OTU96	4.6144	8.03E-04	525	2.80E-04	6
microbiota	OTU199	3.2534	2.90E-06	0	2.72E-04	4
microbiota	OTU310	1.6963	0.00E+00	206	2.56E-04	2
microbiota	OTU155	4.2053	0.00E+00	38	2.74E-04	5
microbiota	OTU462	0.7134	0.00E+00	0	2.49E-04	1
microbiota	OTU67	1.4971	0.00E+00	206	2.59E-04	2

microbiota	OTU149	3.1535	0.00E+00	165	2.72E-04	4
microbiota	OTU88	6.3264	0.00E+00	438	2.70E-04	8
microbiota	OTU306	2.4563	0.00E+00	0	2.77E-04	3
microbiota	OTU431	1.4808	0.00E+00	83	2.62E-04	2
microbiota	OTU31	2.2693	1.70E-03	1	2.72E-04	3
microbiota	OTU113	11.9919	8.87E-01	0	2.78E-04	13
microbiota	OTU184	7.0929	2.98E-01	571	2.82E-04	9
microbiota	OTU54	1.5131	0.00E+00	410	2.67E-04	2
microbiota	OTU36	1.5069	0.00E+00	724	2.69E-04	2
microbiota	OTU33	3.6840	3.30E-05	1783	2.78E-04	5
microbiota	OTU537	2.1752	0.00E+00	419	2.65E-04	3
microbiota	OTU37	5.7052	0.00E+00	701	2.71E-04	7
microbiota	OTU80	3.1265	0.00E+00	319	2.58E-04	4
microbiota	OTU110	2.5859	0.00E+00	0	2.54E-04	3
microbiota	OTU44	1.5417	0.00E+00	0	2.49E-04	2
microbiota	OTU548	0.7617	0.00E+00	0	2.60E-04	1
microbiota	OTU259	6.9736	0.00E+00	0	2.70E-04	8
microbiota	OTU90	5.6544	0.00E+00	89	2.62E-04	7
microbiota	OTU94	6.1328	0.00E+00	0	2.68E-04	7
microbiota	OTU82	6.9276	0.00E+00	0	2.71E-04	8
microbiota	OTU71	5.0943	0.00E+00	0	2.60E-04	6
microbiota	OTU123	6.5341	0.00E+00	1334	2.80E-04	8
microbiota	OTU23	6.4107	0.00E+00	250	2.71E-04	8
microbiota	OTU481	12.3583	6.86E-01	1209	2.84E-04	16
microbiota	OTU200	10.0861	5.85E-01	228	2.80E-04	13
microbiota	OTU223	5.3608	0.00E+00	1052	2.79E-04	7
microbiota	OTU106	1.5258	2.89E-05	0	2.61E-04	2
microbiota	OTU100	3.1714	3.15E-02	151	2.80E-04	4
microbiota	OTU505	3.6588	5.55E-04	1249	2.77E-04	5
microbiota	OTU143	4.9200	1.46E-04	0	2.61E-04	6
microbiota	OTU461	4.0225	1.70E-04	361	2.71E-04	5
microbiota	OTU55	2.2410	1.60E-04	264	2.75E-04	3
microbiota	OTU32	2.1946	5.98E-02	393	2.74E-04	3
microbiota	OTU59	2.1557	3.49E-03	445	2.73E-04	3
microbiota	OTU109	3.4459	0.00E+00	0	2.70E-04	4
microbiota	OTU336	2.5267	0.00E+00	0	2.62E-04	3
microbiota	OTU28	4.0754	0.00E+00	656	2.70E-04	5
microbiota	OTU51	3.0504	0.00E+00	735	2.74E-04	4
microbiota	OTU63	3.0061	8.16E-03	1295	2.84E-04	4
microbiota	OTU52	1.6247	0.00E+00	28	2.64E-04	2
microbiota	OTU46	3.8702	0.00E+00	0	2.71E-04	5
microbiota	OTU158	5.9830	1.00E-05	0	2.71E-04	7
microbiota	OTU451	6.8569	7.30E-05	0	2.74E-04	8
microbiota	OTU81	5.4539	2.66E-05	7	2.70E-04	7
microbiota	OTU410	2.3771	0.00E+00	283	2.61E-04	3
microbiota	OTU239	6.1563	1.16E-05	420	2.70E-04	7
microbiota	OTU616	13.5314	9.51E-01	2	2.79E-04	15
microbiota	OTU150	12.4998	8.58E-01	38	2.79E-04	15
microbiota	OTU101	14.2479	9.73E-01	0	2.79E-04	16
microbiota	OTU34	14.1378	9.67E-01	2	2.79E-04	16

microbiota	OTU45	1.5902	3.38E-02	0	2.69E-04	2
microbiota	OTU64	7.4413	4.06E-01	360	2.81E-04	10
microbiota	OTU488	6.6715	1.96E-02	55	2.75E-04	8
microbiota	OTU318	1.4785	6.61E-04	0	2.61E-04	2
microbiota	OTU30	4.3992	3.28E-03	4	2.69E-04	6
microbiota	OTU137	14.3930	9.10E-01	448	2.83E-04	17
microbiota	OTU91	6.1652	1.89E-01	1129	2.86E-04	8
microbiota	OTU213	3.1404	2.10E-02	1012	2.83E-04	4
microbiota	OTU292	3.7510	1.17E-01	1155	2.82E-04	5
microbiota	OTU72	2.2187	0.00E+00	206	2.66E-04	3
microbiota	OTU16	0.7642	0.00E+00	0	2.55E-04	1
microbiota	OTU185	1.5490	0.00E+00	195	2.63E-04	2
microbiota	OTU22	2.3242	4.24E-05	208	2.65E-04	3
microbiota	OTU169	2.9997	8.36E-04	112	2.66E-04	4
microbiota	OTU41	3.0924	4.08E-04	213	2.71E-04	4
microbiota	OTU335	3.0584	1.04E-01	397	2.78E-04	4
microbiota	OTU7	2.2510	7.30E-03	804	2.76E-04	3
microbiota	OTU115	6.9450	0.00E+00	498	2.76E-04	8
microbiota	OTU476	7.8064	0.00E+00	548	2.79E-04	9
microbiota	OTU56	4.3698	0.00E+00	0	2.66E-04	5
microbiota	OTU73	5.5065	3.79E-04	672	2.75E-04	7
microbiota	OTU29	6.9765	0.00E+00	102	2.69E-04	8
microbiota	OTU221	5.4207	0.00E+00	0	2.69E-04	6
microbiota	OTU139	9.1448	1.33E-04	407	2.78E-04	11
microbiota	OTU517	8.4920	1.22E-04	109	2.78E-04	10
microbiota	OTU164	8.2546	1.36E-04	218	2.80E-04	10
microbiota	OTU119	10.8153	1.53E-04	649	2.82E-04	13
microbiota	OTU487	7.7992	4.65E-05	830	2.81E-04	10
microbiota	OTU68	4.5027	5.12E-05	1319	2.83E-04	6
microbiota	OTU128	12.5128	7.97E-01	671	2.83E-04	15
microbiota	OTU14	0.7102	4.66E-02	0	2.72E-04	1
microbiota	OTU142	5.2058	1.29E-03	1161	2.83E-04	7
microbiota	OTU60	8.4723	0.00E+00	656	2.79E-04	10
microbiota	OTU49	8.7976	9.06E-05	1342	2.83E-04	11
microbiota	OTU86	3.6597	0.00E+00	39	2.75E-04	5
microbiota	OTU103	6.5651	0.00E+00	254	2.77E-04	8
microbiota	OTU356	4.7970	0.00E+00	113	2.73E-04	6
microbiota	OTU76	7.7080	5.70E-05	128	2.77E-04	9
microbiota	OTU10	7.5921	0.00E+00	67	2.75E-04	9
microbiota	OTU39	9.1169	4.10E-05	413	2.79E-04	11
microbiota	OTU62	8.7000	5.96E-04	1928	2.86E-04	11
microbiota	OTU24	7.3691	1.54E-04	219	2.82E-04	9
microbiota	OTU8	9.6333	1.25E-04	515	2.83E-04	12
microbiota	OTU172	9.3616	7.86E-03	557	2.74E-04	12
microbiota	OTU211	10.6318	1.82E-02	1283	2.81E-04	13
microbiota	OTU190	9.8786	9.73E-03	5	2.69E-04	11
microbiota	OTU121	8.0922	7.37E-03	226	2.72E-04	10
microbiota	OTU146	9.1591	7.90E-03	220	2.71E-04	11
microbiota	OTU87	9.3667	1.89E-02	288	2.78E-04	11
microbiota	OTU61	10.5201	1.92E-02	120	2.78E-04	12

microbiota	OTU18	9.7831	9.62E-03	0	2.69E-04	11
microbiota	OTU93	9.0096	9.31E-03	0	2.69E-04	10
microbiota	OTU25	7.9400	7.88E-03	0	2.71E-04	9
microbiota	OTU459	5.6623	0.00E+00	609	2.76E-04	7
microbiota	OTU112	2.3656	2.49E-06	406	2.66E-04	3
microbiota	OTU116	2.1999	7.94E-03	1077	2.81E-04	3
microbiota	OTU58	10.7454	7.65E-01	44	2.79E-04	13
microbiota	OTU21	7.5364	4.44E-01	393	2.78E-04	10
microbiota	OTU5	3.7328	1.28E-01	1076	2.82E-04	5
microbiota	OTU131	7.7418	1.67E-01	2885	2.86E-04	10
microbiota	OTU104	13.0725	8.27E-01	1220	2.85E-04	16
microbiota	OTU151	2.9631	3.22E-05	0	2.75E-04	4
microbiota	OTU153	4.4863	1.62E-03	613	2.79E-04	6
microbiota	OTU424	7.4069	5.59E-04	193	2.78E-04	9
microbiota	OTU321	2.1350	5.52E-04	268	2.72E-04	3
microbiota	OTU84	6.3652	1.31E-04	334	2.77E-04	8
microbiota	OTU40	3.8733	7.88E-03	528	2.77E-04	5
microbiota	OTU53	7.0649	1.26E-04	1367	2.81E-04	9
microbiota	OTU70	6.3940	4.82E-06	1234	2.79E-04	8
microbiota	OTU15	7.1002	7.02E-04	853	2.79E-04	9
microbiota	OTU563	6.5592	6.81E-02	683	2.77E-04	8
microbiota	OTU48	6.2735	8.02E-03	1	2.69E-04	7
microbiota	OTU136	3.0349	0.00E+00	455	2.70E-04	4
microbiota	OTU295	5.4475	8.81E-03	1523	2.77E-04	7
microbiota	OTU43	6.2091	7.12E-04	407	2.71E-04	8
microbiota	OTU4	3.1286	5.73E-04	373	2.71E-04	4
microbiota	OTU649	3.1127	5.23E-04	407	2.71E-04	4
microbiota	OTU2	0.7522	1.60E-05	0	2.60E-04	1
microbiota	OTU170	3.7854	1.55E-03	1303	2.84E-04	5
microbiota	OTU75	3.9611	3.15E-06	385	2.77E-04	5
microbiota	OTU74	3.8021	0.00E+00	761	2.75E-04	5
microbiota	OTU111	4.6397	1.25E-02	1271	2.87E-04	6
microbiota	OTU1	3.1664	8.09E-04	669	2.79E-04	4
microbiota	OTU6	2.2434	5.99E-02	334	2.79E-04	3
microbiota	OTU180	7.2324	9.05E-03	0	2.72E-04	8
microbiota	OTU469	9.5004	9.16E-03	393	2.74E-04	11
microbiota	OTU475	8.0624	9.04E-03	1	2.74E-04	9
microbiota	OTU83	8.0400	9.28E-03	93	2.73E-04	9
microbiota	OTU11	8.3657	4.60E-03	173	2.72E-04	10
microbiota	OTU19	6.7120	9.17E-03	33	2.73E-04	8
microbiota	OTU66	5.2640	9.02E-02	2109	2.85E-04	7
microbiota	OTU12	2.3493	3.22E-04	673	2.74E-04	3
microbiota	OTU99	7.7273	6.94E-04	126	2.69E-04	9
microbiota	OTU134	5.8406	1.58E-04	0	2.60E-04	7
microbiota	OTU135	8.4015	7.28E-04	509	2.70E-04	10
microbiota	OTU394	8.0264	2.22E-04	178	2.64E-04	10
microbiota	OTU35	7.6791	6.73E-04	289	2.69E-04	9
microbiota	OTU17	6.8602	1.52E-04	207	2.63E-04	8
microbiota	OTU97	15.5447	9.77E-01	461	2.80E-04	18
microbiota	OTU129	15.6176	9.83E-01	191	2.82E-04	18

microbiota	OTU65	13.4767	9.13E-01	528	2.83E-04	15
microbiota	OTU397	15.6540	1.00E+00	91	2.80E-04	18
microbiota	OTU378	2.3830	0.00E+00	436	2.64E-04	3
microbiota	OTU20	2.3429	1.28E-02	222	2.74E-04	3
microbiota	OTU105	5.5665	0.00E+00	910	2.78E-04	7
microbiota	OTU50	2.3322	0.00E+00	762	2.63E-04	3
microbiota	OTU38	4.9337	0.00E+00	659	2.69E-04	6
microbiota	OTU107	1.4923	2.07E-04	105	2.66E-04	2
microbiota	OTU191	6.0991	2.06E-03	807	2.76E-04	8
microbiota	Lachnospiraceae	6.7324	5.98E-04	2867	2.86E-04	9
microbiota	L. johnsonii	8.6976	4.33E-05	1005	2.81E-04	11

Table S12. Edges information in multi-omic network analysis given by Fig. 3C.

Node 1	Node 2	Correlation	Relationship	Weight
Serum UA	Isoleucine	-0.9280	negative	0.93
Serum UA	Glycine	-0.8876	negative	0.89
Serum UA	Betaxylose	-0.8852	negative	0.89
Serum UA	Phenylacetate	-0.8804	negative	0.88
Serum UA	Propionate	-0.8697	negative	0.87
Serum UA	Lactate	-0.8464	negative	0.85
Glycine	L. johnsonii	-0.8398	negative	0.84
Alphaglucose	L. johnsonii	-0.8261	negative	0.83
OTU435	OTU549	-0.8163	negative	0.82
Serum UA	Betaglucose	-0.8131	negative	0.81
Valine	OTU6	-0.8000	negative	0.80
Glycine	Lachnospiraceae	-0.7990	negative	0.80
Betaglucose	L. johnsonii	-0.7894	negative	0.79
Taurine	OTU12	-0.7826	negative	0.78
Serum UA	Alphaglucose	-0.7728	negative	0.77
Propionate	L. johnsonii	-0.7710	negative	0.77
Urocanate	OTU107	-0.7629	negative	0.76
Nicotinate	OTU20	-0.7620	negative	0.76
Isoleucine	L. johnsonii	-0.7544	negative	0.75
OTU131	OTU588	-0.7497	negative	0.75
OTU435	OTU130	-0.7379	negative	0.74
OTU111	OTU91	-0.7349	negative	0.73
OTU6	OTU104	-0.7340	negative	0.73
Isoleucine	Lachnospiraceae	-0.7295	negative	0.73
Valine	OTU107	-0.7294	negative	0.73
OTU7	OTU73	-0.7291	negative	0.73
Tryptophan	OTU38	-0.7277	negative	0.73
Phenylacetate	L. johnsonii	-0.7254	negative	0.73
OTU7	OTU172	-0.7238	negative	0.72
OTU213	OTU142	-0.7183	negative	0.72
OTU27	OTU435	-0.7175	negative	0.72
OTU53	OTU68	-0.7148	negative	0.71
OTU62	OTU63	-0.7139	negative	0.71
Propionate	Lachnospiraceae	-0.7108	negative	0.71
OTU14	OTU128	-0.7102	negative	0.71
OTU6	OTU131	-0.7093	negative	0.71
OTU87	OTU172	0.7079	positive	0.71
OTU64	OTU34	0.7079	positive	0.71
OTU66	OTU184	0.7081	positive	0.71
OTU5	OTU116	0.7082	positive	0.71
Betagalactose	OTU19	0.7084	positive	0.71
OTU21	OTU129	0.7085	positive	0.71
Serum UA	Lachnospiraceae	0.7086	positive	0.71
OTU66	OTU435	0.7088	positive	0.71
OTU28	OTU505	0.7088	positive	0.71
OTU62	OTU49	0.7088	positive	0.71
OTU31	OTU321	0.7097	positive	0.71

OTU65	OTU131	0.7098	positive	0.71
OTU21	OTU64	0.7100	positive	0.71
OTU37	OTU123	0.7104	positive	0.71
OTU108	OTU469	0.7106	positive	0.71
OTU30	OTU190	0.7109	positive	0.71
OTU59	OTU32	0.7111	positive	0.71
Lachnospiraceae	OTU588	0.7111	positive	0.71
OTU397	OTU184	0.7112	positive	0.71
OTU49	OTU115	0.7113	positive	0.71
OTU46	OTU115	0.7115	positive	0.71
OTU44	OTU80	0.7118	positive	0.71
OTU82	OTU223	0.7121	positive	0.71
OTU15	OTU321	0.7122	positive	0.71
OTU24	OTU142	0.7128	positive	0.71
OTU39	OTU105	0.7130	positive	0.71
OTU33	OTU476	0.7131	positive	0.71
OTU321	OTU121	0.7131	positive	0.71
OTU59	OTU461	0.7132	positive	0.71
OTU67	OTU462	0.7134	positive	0.71
OTU53	OTU151	0.7135	positive	0.71
OTU51	OTU72	0.7135	positive	0.71
OTU39	OTU142	0.7136	positive	0.71
OTU30	OTU61	0.7142	positive	0.71
OTU146	OTU318	0.7143	positive	0.71
OTU200	OTU616	0.7149	positive	0.71
OTU32	OTU97	0.7150	positive	0.71
OTU335	OTU481	0.7156	positive	0.72
OTU356	OTU86	0.7157	positive	0.72
OTU21	OTU34	0.7159	positive	0.72
OTU41	OTU191	0.7159	positive	0.72
OTU130	OTU119	0.7160	positive	0.72
OTU74	OTU75	0.7162	positive	0.72
OTU43	OTU99	0.7162	positive	0.72
OTU235	OTU170	0.7162	positive	0.72
OTU114	OTU537	0.7162	positive	0.72
OTU137	OTU481	0.7164	positive	0.72
OTU43	OTU106	0.7167	positive	0.72
OTU150	OTU200	0.7168	positive	0.72
OTU469	OTU169	0.7171	positive	0.72
OTU8	OTU86	0.7173	positive	0.72
OTU164	OTU451	0.7180	positive	0.72
OTU5	OTU104	0.7187	positive	0.72
OTU109	OTU505	0.7188	positive	0.72
Lachnospiraceae	OTU123	0.7191	positive	0.72
OTU476	OTU49	0.7191	positive	0.72
OTU53	OTU153	0.7196	positive	0.72
OTU73	OTU60	0.7196	positive	0.72
OTU131	OTU211	0.7199	positive	0.72
OTU33	OTU60	0.7201	positive	0.72
OTU170	OTU588	0.7216	positive	0.72

OTU21	OTU616	0.7218	positive	0.72
OTU62	OTU517	0.7220	positive	0.72
OTU424	OTU151	0.7225	positive	0.72
OTU40	OTU153	0.7227	positive	0.72
Alphaarabinose	OTU129	0.7229	positive	0.72
OTU62	OTU142	0.7229	positive	0.72
OTU487	OTU119	0.7234	positive	0.72
OTU64	OTU397	0.7236	positive	0.72
Betagalactose	OTU11	0.7238	positive	0.72
OTU90	OTU537	0.7240	positive	0.72
Butyrate	Lactate	0.7241	positive	0.72
OTU29	OTU52	0.7246	positive	0.72
OTU8	OTU10	0.7248	positive	0.72
OTU23	OTU223	0.7254	positive	0.73
OTU36	OTU50	0.7257	positive	0.73
OTU58	OTU150	0.7260	positive	0.73
OTU64	OTU101	0.7263	positive	0.73
OTU35	OTU295	0.7264	positive	0.73
OTU410	OTU80	0.7265	positive	0.73
OTU84	OTU153	0.7270	positive	0.73
OTU88	OTU149	0.7270	positive	0.73
OTU21	OTU101	0.7272	positive	0.73
Choline	OTU17	0.7273	positive	0.73
OTU118	OTU235	0.7276	positive	0.73
OTU86	OTU105	0.7278	positive	0.73
OTU15	OTU84	0.7279	positive	0.73
OTU21	OTU397	0.7281	positive	0.73
OTU111	OTU235	0.7282	positive	0.73
Alphaarabinose	OTU397	0.7286	positive	0.73
OTU51	OTU70	0.7290	positive	0.73
OTU53	OTU84	0.7294	positive	0.73
OTU30	OTU18	0.7294	positive	0.73
OTU139	OTU158	0.7297	positive	0.73
Alphaarabinose	OTU65	0.7299	positive	0.73
OTU22	OTU169	0.7300	positive	0.73
OTU34	OTU200	0.7304	positive	0.73
OTU19	OTU11	0.7306	positive	0.73
OTU25	OTU172	0.7307	positive	0.73
OTU40	OTU424	0.7309	positive	0.73
OTU59	OTU55	0.7314	positive	0.73
Propionate	Alphaglucose	0.7315	positive	0.73
OTU33	OTU54	0.7318	positive	0.73
OTU394	OTU134	0.7320	positive	0.73
Betaxylose	OTU105	0.7322	positive	0.73
OTU64	OTU481	0.7322	positive	0.73
OTU129	OTU91	0.7328	positive	0.73
OTU96	OTU517	0.7328	positive	0.73
OTU64	OTU97	0.7330	positive	0.73
OTU81	OTU164	0.7332	positive	0.73
OTU43	OTU35	0.7334	positive	0.73

OTU68	OTU164	0.7336	positive	0.73
OTU62	OTU487	0.7337	positive	0.73
OTU103	OTU487	0.7337	positive	0.73
OTU58	OTU137	0.7339	positive	0.73
OTU292	OTU137	0.7341	positive	0.73
OTU20	OTU488	0.7342	positive	0.73
OTU11	OTU191	0.7344	positive	0.73
OTU101	OTU200	0.7345	positive	0.73
OTU191	OTU505	0.7346	positive	0.73
OTU549	OTU166	0.7348	positive	0.73
OTU37	OTU537	0.7350	positive	0.73
OTU116	OTU505	0.7351	positive	0.74
OTU469	OTU191	0.7352	positive	0.74
OTU114	OTU135	0.7353	positive	0.74
OTU5	OTU40	0.7356	positive	0.74
OTU30	OTU172	0.7359	positive	0.74
OTU96	OTU451	0.7362	positive	0.74
OTU125	OTU191	0.7363	positive	0.74
OTU431	OTU175	0.7365	positive	0.74
OTU83	OTU69	0.7372	positive	0.74
Lachnospiraceae	OTU223	0.7373	positive	0.74
OTU33	OTU170	0.7379	positive	0.74
Creatine	Taurine	0.7380	positive	0.74
Betagalactose	OTU66	0.7382	positive	0.74
OTU87	OTU121	0.7387	positive	0.74
OTU146	OTU121	0.7388	positive	0.74
OTU88	OTU459	0.7388	positive	0.74
Choline	OTU134	0.7390	positive	0.74
OTU86	OTU49	0.7392	positive	0.74
OTU81	OTU158	0.7394	positive	0.74
OTU1	OTU74	0.7401	positive	0.74
OTU292	OTU91	0.7402	positive	0.74
OTU38	OTU72	0.7411	positive	0.74
OTU68	OTU119	0.7417	positive	0.74
OTU34	OTU481	0.7418	positive	0.74
OTU97	OTU481	0.7425	positive	0.74
OTU121	OTU172	0.7427	positive	0.74
OTU113	OTU481	0.7435	positive	0.74
L. johnsonii	OTU55	0.7438	positive	0.74
OTU21	OTU97	0.7440	positive	0.74
Glycine	Betaxylose	0.7442	positive	0.74
OTU28	OTU431	0.7444	positive	0.74
OTU356	OTU88	0.7446	positive	0.74
OTU394	OTU143	0.7446	positive	0.74
OTU30	OTU146	0.7447	positive	0.74
OTU475	OTU191	0.7447	positive	0.74
OTU8	OTU24	0.7451	positive	0.75
OTU74	OTU136	0.7452	positive	0.75
OTU53	OTU199	0.7456	positive	0.75
OTU24	OTU68	0.7456	positive	0.75

OTU104	OTU137	0.7456	positive	0.75
OTU4	OTU394	0.7457	positive	0.75
OTU104	OTU131	0.7461	positive	0.75
OTU76	OTU142	0.7461	positive	0.75
OTU65	OTU481	0.7470	positive	0.75
OTU223	OTU259	0.7472	positive	0.75
OTU8	OTU356	0.7473	positive	0.75
OTU292	OTU128	0.7478	positive	0.75
OTU62	OTU68	0.7479	positive	0.75
OTU24	OTU517	0.7481	positive	0.75
OTU292	OTU295	0.7483	positive	0.75
OTU616	OTU481	0.7486	positive	0.75
OTU136	OTU461	0.7486	positive	0.75
L. johnsonii	OTU75	0.7487	positive	0.75
OTU61	OTU131	0.7492	positive	0.75
OTU397	OTU200	0.7503	positive	0.75
OTU91	OTU184	0.7504	positive	0.75
Urocanate	Betaxylose	0.7504	positive	0.75
OTU48	OTU563	0.7505	positive	0.75
OTU153	OTU211	0.7511	positive	0.75
OTU101	OTU481	0.7513	positive	0.75
OTU146	OTU211	0.7514	positive	0.75
OTU2	OTU649	0.7522	positive	0.75
Choline	OTU99	0.7523	positive	0.75
OTU57	OTU112	0.7539	positive	0.75
OTU200	OTU91	0.7540	positive	0.75
OTU39	OTU459	0.7544	positive	0.75
OTU295	OTU99	0.7544	positive	0.75
OTU1	OTU111	0.7547	positive	0.75
OTU15	OTU70	0.7548	positive	0.75
OTU185	OTU136	0.7550	positive	0.75
OTU149	OTU487	0.7552	positive	0.76
Lachnospiraceae	OTU306	0.7560	positive	0.76
OTU63	OTU116	0.7566	positive	0.76
OTU129	OTU184	0.7569	positive	0.76
OTU96	OTU164	0.7577	positive	0.76
OTU424	OTU153	0.7578	positive	0.76
OTU31	OTU172	0.7580	positive	0.76
OTU12	OTU112	0.7581	positive	0.76
OTU96	OTU119	0.7582	positive	0.76
OTU15	OTU151	0.7595	positive	0.76
OTU39	OTU86	0.7598	positive	0.76
OTU64	OTU335	0.7609	positive	0.76
OTU129	OTU481	0.7609	positive	0.76
OTU397	OTU481	0.7610	positive	0.76
Glycine	Lactate	0.7611	positive	0.76
OTU24	OTU487	0.7611	positive	0.76
Lactate	OTU378	0.7612	positive	0.76
OTU97	OTU200	0.7615	positive	0.76
OTU41	OTU505	0.7615	positive	0.76

OTU23	OTU548	0.7617	positive	0.76
OTU424	OTU199	0.7617	positive	0.76
OTU100	OTU184	0.7622	positive	0.76
OTU103	OTU88	0.7627	positive	0.76
OTU15	OTU649	0.7628	positive	0.76
OTU63	OTU66	0.7638	positive	0.76
OTU16	OTU72	0.7642	positive	0.76
OTU30	OTU318	0.7642	positive	0.76
Alphaarabinose	OTU97	0.7643	positive	0.76
OTU55	OTU75	0.7658	positive	0.77
OTU43	OTU135	0.7658	positive	0.77
OTU84	OTU166	0.7661	positive	0.77
OTU49	OTU142	0.7664	positive	0.77
OTU70	OTU151	0.7675	positive	0.77
OTU32	OTU45	0.7685	positive	0.77
OTU5	OTU58	0.7691	positive	0.77
OTU87	OTU131	0.7695	positive	0.77
Taurine	OTU66	0.7701	positive	0.77
Betaxylose	Propionate	0.7701	positive	0.77
OTU111	OTU96	0.7703	positive	0.77
OTU487	OTU139	0.7703	positive	0.77
OTU93	OTU488	0.7703	positive	0.77
OTU25	OTU211	0.7713	positive	0.77
OTU63	OTU100	0.7717	positive	0.77
OTU50	OTU176	0.7718	positive	0.77
OTU64	OTU66	0.7719	positive	0.77
OTU70	OTU123	0.7725	positive	0.77
OTU81	OTU119	0.7728	positive	0.77
OTU239	OTU410	0.7734	positive	0.77
OTU108	OTU169	0.7738	positive	0.77
Betagalactose	OTU83	0.7739	positive	0.77
OTU221	OTU73	0.7743	positive	0.77
OTU81	OTU139	0.7766	positive	0.78
OTU378	OTU80	0.7770	positive	0.78
OTU100	OTU91	0.7783	positive	0.78
OTU137	OTU184	0.7784	positive	0.78
OTU11	OTU169	0.7788	positive	0.78
OTU200	OTU137	0.7795	positive	0.78
OTU46	OTU476	0.7798	positive	0.78
OTU29	OTU46	0.7800	positive	0.78
OTU292	OTU213	0.7806	positive	0.78
OTU33	OTU36	0.7812	positive	0.78
OTU10	OTU149	0.7812	positive	0.78
OTU54	OTU310	0.7813	positive	0.78
OTU103	OTU105	0.7815	positive	0.78
OTU8	OTU60	0.7821	positive	0.78
OTU10	OTU487	0.7821	positive	0.78
OTU70	OTU155	0.7825	positive	0.78
OTU94	OTU90	0.7827	positive	0.78
OTU104	OTU150	0.7829	positive	0.78

OTU146	OTU172	0.7830	positive	0.78
OTU8	OTU62	0.7832	positive	0.78
OTU111	OTU170	0.7833	positive	0.78
OTU67	OTU88	0.7837	positive	0.78
OTU335	OTU200	0.7838	positive	0.78
OTU74	OTU461	0.7842	positive	0.78
OTU129	OTU200	0.7853	positive	0.79
OTU64	OTU150	0.7853	positive	0.79
OTU104	OTU481	0.7856	positive	0.79
OTU563	OTU184	0.7859	positive	0.79
OTU4	OTU136	0.7861	positive	0.79
OTU4	OTU295	0.7863	positive	0.79
OTU73	OTU46	0.7865	positive	0.79
OTU27	OTU130	0.7869	positive	0.79
Choline	OTU35	0.7879	positive	0.79
OTU58	OTU128	0.7883	positive	0.79
OTU164	OTU517	0.7884	positive	0.79
OTU145	OTU459	0.7888	positive	0.79
OTU23	OTU123	0.7888	positive	0.79
OTU649	OTU175	0.7891	positive	0.79
OTU73	OTU115	0.7895	positive	0.79
OTU64	OTU200	0.7902	positive	0.79
Choline	OTU394	0.7911	positive	0.79
OTU27	OTU549	0.7917	positive	0.79
OTU8	OTU103	0.7930	positive	0.79
OTU84	OTU549	0.7933	positive	0.79
OTU22	OTU185	0.7940	positive	0.79
OTU23	OTU94	0.7940	positive	0.79
OTU38	OTU51	0.7947	positive	0.79
OTU239	OTU81	0.7955	positive	0.80
OTU235	OTU588	0.7956	positive	0.80
OTU10	OTU459	0.7970	positive	0.80
OTU123	OTU223	0.7971	positive	0.80
OTU28	OTU336	0.7975	positive	0.80
OTU131	OTU128	0.7976	positive	0.80
Betagalactose	OTU469	0.7981	positive	0.80
OTU7	OTU335	0.7981	positive	0.80
OTU62	OTU139	0.7984	positive	0.80
OTU43	OTU394	0.7986	positive	0.80
OTU150	OTU128	0.7988	positive	0.80
OTU563	OTU481	0.7995	positive	0.80
OTU8	OTU39	0.7998	positive	0.80
OTU22	OTU41	0.8002	positive	0.80
OTU134	OTU143	0.8004	positive	0.80
OTU5	OTU21	0.8011	positive	0.80
OTU90	OTU259	0.8014	positive	0.80
OTU31	OTU211	0.8017	positive	0.80
OTU66	OTU481	0.8033	positive	0.80
OTU475	OTU563	0.8034	positive	0.80
OTU15	OTU53	0.8036	positive	0.80

Hypoxanthine	Betaxylose	0.8046	positive	0.80
OTU17	OTU394	0.8051	positive	0.81
Betagalactose	OTU475	0.8052	positive	0.81
OTU61	OTU121	0.8061	positive	0.81
OTU39	OTU49	0.8062	positive	0.81
OTU295	OTU135	0.8065	positive	0.81
OTU239	OTU139	0.8075	positive	0.81
OTU76	OTU88	0.8076	positive	0.81
OTU37	OTU90	0.8078	positive	0.81
OTU15	OTU153	0.8080	positive	0.81
OTU12	OTU459	0.8086	positive	0.81
OTU40	OTU649	0.8087	positive	0.81
Choline	OTU135	0.8087	positive	0.81
OTU23	OTU90	0.8090	positive	0.81
OTU106	OTU394	0.8091	positive	0.81
OTU81	OTU451	0.8092	positive	0.81
OTU4	OTU43	0.8105	positive	0.81
OTU563	OTU469	0.8106	positive	0.81
OTU17	OTU143	0.8107	positive	0.81
OTU43	OTU295	0.8109	positive	0.81
OTU49	OTU60	0.8122	positive	0.81
OTU135	OTU143	0.8123	positive	0.81
OTU46	OTU60	0.8124	positive	0.81
OTU58	OTU97	0.8128	positive	0.81
OTU51	OTU223	0.8132	positive	0.81
Phenylacetate	Butyrate	0.8138	positive	0.81
OTU18	OTU488	0.8145	positive	0.81
OTU11	OTU41	0.8147	positive	0.81
OTU295	OTU213	0.8148	positive	0.81
OTU27	OTU235	0.8149	positive	0.81
L. johnsonii	OTU306	0.8150	positive	0.82
OTU58	OTU129	0.8156	positive	0.82
OTU74	OTU160	0.8165	positive	0.82
OTU18	OTU172	0.8176	positive	0.82
OTU176	OTU157	0.8179	positive	0.82
OTU87	OTU211	0.8187	positive	0.82
OTU68	OTU487	0.8191	positive	0.82
OTU18	OTU121	0.8197	positive	0.82
Isoleucine	Betaxylose	0.8198	positive	0.82
OTU58	OTU104	0.8204	positive	0.82
OTU73	OTU476	0.8211	positive	0.82
OTU21	OTU45	0.8217	positive	0.82
OTU61	OTU172	0.8227	positive	0.82
Serum UA	L. johnsonii	0.8630	positive	0.86
OTU71	OTU259	0.8234	positive	0.82
Betagalactose	OTU180	0.8246	positive	0.82
OTU76	OTU356	0.8246	positive	0.82
OTU8	OTU142	0.8256	positive	0.83
OTU35	OTU143	0.8263	positive	0.83
OTU1	OTU170	0.8264	positive	0.83

OTU213	OTU91	0.8267	positive	0.83
OTU81	OTU517	0.8271	positive	0.83
OTU70	OTU223	0.8285	positive	0.83
OTU76	OTU105	0.8286	positive	0.83
OTU71	OTU94	0.8288	positive	0.83
OTU104	OTU129	0.8293	positive	0.83
OTU44	OTU110	0.8299	positive	0.83
OTU157	OTU124	0.8310	positive	0.83
Betagalactose	OTU191	0.8314	positive	0.83
OTU488	OTU190	0.8323	positive	0.83
OTU139	OTU451	0.8323	positive	0.83
OTU150	OTU137	0.8326	positive	0.83
OTU23	OTU71	0.8333	positive	0.83
OTU34	OTU104	0.8336	positive	0.83
OTU18	OTU211	0.8336	positive	0.83
OTU104	OTU128	0.8337	positive	0.83
OTU95	OTU160	0.8347	positive	0.83
Tryptophan	OTU50	0.8347	positive	0.83
OTU93	OTU172	0.8348	positive	0.83
OTU394	OTU99	0.8350	positive	0.83
Betaglucose	Betagalactose	0.8354	positive	0.84
OTU487	OTU164	0.8360	positive	0.84
OTU155	OTU199	0.8366	positive	0.84
OTU10	OTU356	0.8376	positive	0.84
OTU563	OTU180	0.8385	positive	0.84
OTU58	OTU101	0.8386	positive	0.84
OTU24	OTU139	0.8387	positive	0.84
OTU82	OTU90	0.8392	positive	0.84
OTU97	OTU128	0.8399	positive	0.84
OTU76	OTU49	0.8401	positive	0.84
OTU19	OTU180	0.8403	positive	0.84
OTU53	OTU155	0.8421	positive	0.84
OTU83	OTU563	0.8424	positive	0.84
OTU488	OTU211	0.8426	positive	0.84
OTU397	OTU104	0.8428	positive	0.84
Phenylacetate	Glycine	0.8431	positive	0.84
Butyrate	Nicotinate	0.8434	positive	0.84
OTU37	OTU259	0.8445	positive	0.84
OTU378	OTU110	0.8449	positive	0.84
OTU397	OTU58	0.8450	positive	0.85
OTU1	OTU75	0.8452	positive	0.85
OTU121	OTU190	0.8456	positive	0.85
OTU150	OTU113	0.8464	positive	0.85
OTU20	OTU131	0.8466	positive	0.85
OTU29	OTU56	0.8470	positive	0.85
OTU19	OTU48	0.8474	positive	0.85
OTU91	OTU137	0.8478	positive	0.85
OTU61	OTU211	0.8482	positive	0.85
OTU37	OTU82	0.8488	positive	0.85
OTU394	OTU135	0.8491	positive	0.85

OTU23	OTU82	0.8492	positive	0.85
OTU23	OTU259	0.8493	positive	0.85
OTU39	OTU88	0.8494	positive	0.85
OTU190	OTU172	0.8500	positive	0.85
OTU10	OTU103	0.8503	positive	0.85
OTU397	OTU128	0.8526	positive	0.85
OTU17	OTU112	0.8536	positive	0.85
OTU221	OTU56	0.8539	positive	0.85
OTU58	OTU65	0.8539	positive	0.85
OTU211	OTU172	0.8545	positive	0.85
OTU84	OTU155	0.8549	positive	0.85
OTU104	OTU97	0.8559	positive	0.86
Betaxylose	Lactate	0.8562	positive	0.86
OTU71	OTU82	0.8562	positive	0.86
OTU84	OTU424	0.8570	positive	0.86
OTU128	OTU137	0.8571	positive	0.86
OTU43	OTU461	0.8571	positive	0.86
OTU104	OTU101	0.8575	positive	0.86
Glycine	Propionate	0.8576	positive	0.86
OTU21	OTU150	0.8581	positive	0.86
OTU129	OTU150	0.8581	positive	0.86
OTU38	OTU336	0.8588	positive	0.86
OTU96	OTU139	0.8592	positive	0.86
OTU100	OTU69	0.8592	positive	0.86
OTU121	OTU211	0.8597	positive	0.86
L. johnsonii	Lachnospiraceae	0.8609	positive	0.86
OTU34	OTU128	0.8609	positive	0.86
OTU61	OTU488	0.8615	positive	0.86
OTU129	OTU128	0.8618	positive	0.86
OTU37	OTU71	0.8624	positive	0.86
OTU103	OTU49	0.8630	positive	0.86
OTU8	OTU76	0.8633	positive	0.86
OTU97	OTU150	0.8637	positive	0.86
Glycine	Isoleucine	0.8648	positive	0.86
OTU517	OTU139	0.8655	positive	0.87
Betaglucose	OTU191	0.8666	positive	0.87
OTU135	OTU134	0.8675	positive	0.87
OTU111	OTU588	0.8683	positive	0.87
OTU87	OTU146	0.8693	positive	0.87
OTU239	OTU119	0.8694	positive	0.87
OTU34	OTU150	0.8698	positive	0.87
OTU119	OTU158	0.8702	positive	0.87
OTU336	OTU109	0.8704	positive	0.87
OTU62	OTU119	0.8709	positive	0.87
Urocanate	Hypoxanthine	0.8720	positive	0.87
Phenylacetate	Isoleucine	0.8721	positive	0.87
OTU87	OTU488	0.8722	positive	0.87
OTU19	OTU475	0.8735	positive	0.87
OTU128	OTU113	0.8747	positive	0.87
OTU15	OTU40	0.8754	positive	0.88

Phenylacetate	Betaxylose	0.8767	positive	0.88
OTU200	OTU481	0.8770	positive	0.88
OTU410	OTU158	0.8772	positive	0.88
OTU70	OTU424	0.8773	positive	0.88
OTU35	OTU134	0.8775	positive	0.88
OTU119	OTU451	0.8790	positive	0.88
OTU25	OTU87	0.8793	positive	0.88
OTU25	OTU121	0.8794	positive	0.88
OTU190	OTU211	0.8804	positive	0.88
OTU17	OTU135	0.8810	positive	0.88
OTU49	OTU105	0.8815	positive	0.88
OTU70	OTU53	0.8818	positive	0.88
OTU19	OTU469	0.8832	positive	0.88
OTU93	OTU87	0.8834	positive	0.88
OTU65	OTU150	0.8841	positive	0.88
OTU93	OTU146	0.8844	positive	0.88
OTU487	OTU459	0.8846	positive	0.88
OTU306	OTU75	0.8853	positive	0.89
Isoleucine	Lactate	0.8858	positive	0.89
OTU56	OTU476	0.8862	positive	0.89
OTU29	OTU73	0.8864	positive	0.89
OTU123	OTU82	0.8874	positive	0.89
OTU397	OTU150	0.8881	positive	0.89
OTU101	OTU128	0.8888	positive	0.89
OTU56	OTU60	0.8891	positive	0.89
OTU155	OTU424	0.8891	positive	0.89
OTU119	OTU164	0.8897	positive	0.89
OTU76	OTU103	0.8897	positive	0.89
OTU38	OTU28	0.8898	positive	0.89
OTU149	OTU459	0.8901	positive	0.89
OTU71	OTU90	0.8902	positive	0.89
OTU104	OTU616	0.8904	positive	0.89
OTU11	OTU48	0.8904	positive	0.89
OTU34	OTU58	0.8907	positive	0.89
OTU150	OTU616	0.8907	positive	0.89
OTU39	OTU103	0.8912	positive	0.89
OTU137	OTU113	0.8914	positive	0.89
OTU25	OTU146	0.8921	positive	0.89
OTU24	OTU119	0.8921	positive	0.89
OTU11	OTU83	0.8935	positive	0.89
OTU56	OTU115	0.8935	positive	0.89
OTU65	OTU128	0.8940	positive	0.89
Propionate	Lactate	0.8956	positive	0.90
OTU15	OTU424	0.8960	positive	0.90
OTU37	OTU94	0.8963	positive	0.90
OTU104	OTU113	0.8977	positive	0.90
OTU65	OTU104	0.8981	positive	0.90
OTU101	OTU150	0.8984	positive	0.90
OTU93	OTU211	0.8986	positive	0.90
OTU118	OTU52	0.9001	positive	0.90

OTU19	OTU83	0.9001	positive	0.90
OTU29	OTU60	0.9018	positive	0.90
OTU8	OTU105	0.9020	positive	0.90
OTU451	OTU158	0.9043	positive	0.90
OTU62	OTU164	0.9043	positive	0.90
OTU119	OTU139	0.9045	positive	0.90
OTU123	OTU94	0.9046	positive	0.90
OTU48	OTU180	0.9054	positive	0.91
OTU616	OTU128	0.9066	positive	0.91
OTU616	OTU137	0.9069	positive	0.91
OTU200	OTU184	0.9080	positive	0.91
OTU84	OTU199	0.9095	positive	0.91
OTU17	OTU134	0.9102	positive	0.91
OTU65	OTU137	0.9102	positive	0.91
OTU110	OTU80	0.9111	positive	0.91
OTU10	OTU88	0.9125	positive	0.91
OTU61	OTU146	0.9127	positive	0.91
Phenylacetate	Lactate	0.9139	positive	0.91
OTU134	OTU99	0.9139	positive	0.91
OTU34	OTU137	0.9142	positive	0.91
OTU53	OTU424	0.9145	positive	0.91
OTU58	OTU616	0.9148	positive	0.91
OTU102	OTU310	0.9150	positive	0.91
OTU35	OTU394	0.9161	positive	0.92
OTU517	OTU158	0.9170	positive	0.92
OTU97	OTU137	0.9173	positive	0.92
OTU35	OTU135	0.9180	positive	0.92
OTU11	OTU180	0.9183	positive	0.92
OTU461	OTU160	0.9193	positive	0.92
OTU38	OTU109	0.9217	positive	0.92
OTU221	OTU29	0.9233	positive	0.92
Phenylacetate	Propionate	0.9247	positive	0.92
OTU17	OTU99	0.9257	positive	0.93
OTU99	OTU143	0.9258	positive	0.93
OTU39	OTU356	0.9270	positive	0.93
OTU119	OTU517	0.9274	positive	0.93
OTU397	OTU137	0.9282	positive	0.93
OTU19	OTU563	0.9284	positive	0.93
OTU221	OTU60	0.9292	positive	0.93
OTU146	OTU190	0.9294	positive	0.93
OTU24	OTU164	0.9317	positive	0.93
OTU184	OTU481	0.9321	positive	0.93
OTU101	OTU137	0.9326	positive	0.93
OTU93	OTU61	0.9347	positive	0.93
OTU28	OTU109	0.9349	positive	0.93
OTU25	OTU61	0.9353	positive	0.94
OTU11	OTU475	0.9358	positive	0.94
OTU58	OTU113	0.9363	positive	0.94
OTU87	OTU190	0.9371	positive	0.94
OTU60	OTU115	0.9386	positive	0.94

OTU18	OTU146	0.9388	positive	0.94
Isoleucine	Propionate	0.9408	positive	0.94
OTU18	OTU87	0.9415	positive	0.94
OTU131	OTU488	0.9439	positive	0.94
OTU93	OTU18	0.9449	positive	0.94
OTU239	OTU158	0.9453	positive	0.95
OTU11	OTU469	0.9453	positive	0.95
OTU29	OTU115	0.9458	positive	0.95
OTU25	OTU190	0.9464	positive	0.95
OTU17	OTU35	0.9467	positive	0.95
OTU35	OTU99	0.9469	positive	0.95
OTU48	OTU475	0.9480	positive	0.95
OTU93	OTU121	0.9484	positive	0.95
OTU61	OTU87	0.9491	positive	0.95
OTU8	OTU49	0.9497	positive	0.95
OTU39	OTU10	0.9506	positive	0.95
OTU25	OTU93	0.9510	positive	0.95
OTU97	OTU113	0.9513	positive	0.95
OTU39	OTU76	0.9519	positive	0.95
OTU82	OTU94	0.9537	positive	0.95
OTU123	OTU259	0.9541	positive	0.95
OTU25	OTU18	0.9547	positive	0.95
OTU97	OTU616	0.9555	positive	0.96
OTU10	OTU76	0.9560	positive	0.96
OTU129	OTU113	0.9561	positive	0.96
OTU135	OTU99	0.9572	positive	0.96
OTU48	OTU469	0.9573	positive	0.96
OTU93	OTU190	0.9592	positive	0.96
OTU83	OTU180	0.9612	positive	0.96
OTU129	OTU616	0.9613	positive	0.96
OTU164	OTU139	0.9621	positive	0.96
OTU129	OTU137	0.9669	positive	0.97
OTU476	OTU60	0.9673	positive	0.97
OTU29	OTU476	0.9674	positive	0.97
OTU469	OTU180	0.9676	positive	0.97
OTU65	OTU97	0.9684	positive	0.97
OTU65	OTU129	0.9688	positive	0.97
OTU221	OTU476	0.9688	positive	0.97
OTU101	OTU113	0.9697	positive	0.97
OTU34	OTU97	0.9706	positive	0.97
OTU221	OTU115	0.9711	positive	0.97
OTU397	OTU113	0.9726	positive	0.97
OTU94	OTU259	0.9727	positive	0.97
OTU48	OTU83	0.9745	positive	0.97
OTU239	OTU517	0.9755	positive	0.98
OTU34	OTU65	0.9755	positive	0.98
OTU475	OTU180	0.9765	positive	0.98
OTU34	OTU129	0.9768	positive	0.98
OTU83	OTU475	0.9785	positive	0.98
OTU397	OTU616	0.9786	positive	0.98

OTU83	OTU469	0.9787	positive	0.98
OTU65	OTU113	0.9789	positive	0.98
OTU82	OTU259	0.9811	positive	0.98
OTU65	OTU616	0.9815	positive	0.98
OTU101	OTU97	0.9815	positive	0.98
OTU34	OTU113	0.9819	positive	0.98
OTU101	OTU616	0.9821	positive	0.98
OTU397	OTU97	0.9827	positive	0.98
OTU476	OTU115	0.9836	positive	0.98
OTU129	OTU101	0.9848	positive	0.98
OTU129	OTU97	0.9849	positive	0.98
OTU397	OTU129	0.9858	positive	0.99
OTU397	OTU65	0.9864	positive	0.99
OTU34	OTU616	0.9864	positive	0.99
OTU517	OTU451	0.9882	positive	0.99
OTU34	OTU101	0.9888	positive	0.99
OTU239	OTU451	0.9897	positive	0.99
OTU65	OTU101	0.9900	positive	0.99
OTU616	OTU113	0.9914	positive	0.99
OTU397	OTU34	0.9926	positive	0.99
OTU61	OTU190	0.9927	positive	0.99
OTU18	OTU61	0.9937	positive	0.99
OTU24	OTU62	0.9939	positive	0.99
OTU18	OTU190	0.9946	positive	0.99
OTU397	OTU101	0.9957	positive	1.00
OTU475	OTU469	0.9967	positive	1.00
