# Supplementary material for: Lactiplantibacillus plantarum enables blood urate control in mice through degradation of nucleosides in gastrointestinal tract
Source: Microbiome. 2023 Jul 19;11:153. doi: 10.1186/s40168-023-01605-y (PMC10354915; doi:10.1186/s40168-023-01605-y)
Supplement: Supplementary file 3 — Additional file 2: Figure S1. Changes in metabolic parameters due to high nucleosides diet and L. plantarum supplementation in mice. Supplementary Fig. 2. Changes in gut microbiota due to high nucleosides diet and L. plantarum supplementation in mice. Supplementary Fig. 3. L. plantarum affecting the gut metabolites. Supplementary Fig. 4. Metagenomic functional annotation and classification of L. plantarum in KEGG (A) and in COG (B). Supplementary Fig. 5. Identification of production of inosine hydrolyzed by L. plantarum. Supplementary Fig. 6. Established Caco-2 cell monolayer for nucleosides and nucleobases transport. Supplementary Fig. 7. Gene sequences alignment of RihA–C from L. plantarum with other organisms. [file 40168_2023_1605_MOESM2_ESM.docx]

**Supplementary Figures**


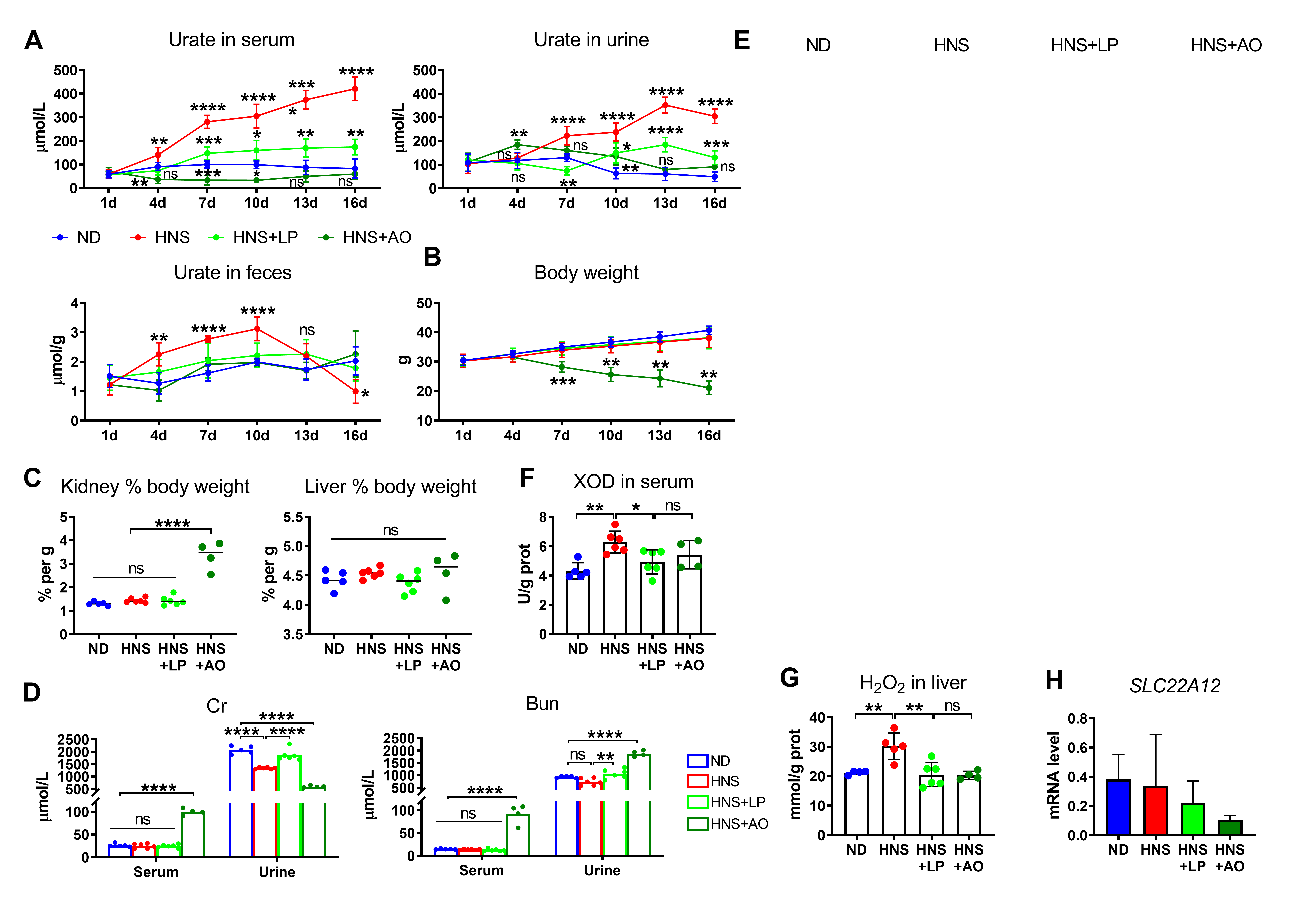


**Figure S1. Changes in metabolic parameters due to high nucleosides diet and *L. plantarum* supplementation in mice. A** Urate levels in serum, urine and feces during 16 days. There are significant differences between HNS mice and ND mice. Changes of body weight (**B**), ratio of kidney (left panel in **C**) and liver weight to body weight (right panel in **C**). **D** Renal function indexes of creatinine (Cr) (left panel) and urea nitrogen (Bun) concentrations (right panel) in serum and urine. **E** Hematoxylin and eosin **(**H&E)-staining images of liver (upper panel), kidney (middle panel) and intestine (lower panel). Arrows indicate inflammatory cell infiltration (green), lumenal crystals (yellow) and intratubular abscess (red), respectively. XOD activity in serum (**F**) and H_2_O_2_ concentrations in liver (**G**) of mice at 16 days after euthanasia. **H** qRT-PCR analysis of mRNA levels of urate transporter *SCL22A12* in kidney. ND, normal diet; HNS, high-nucleoside diet; HNS+LP, high-nucleoside diet supplemented *L. plantarum*; HNS+AO, high-nucleoside diet plus allopurinol treatment. Graphs show the mean ± s.d. (A, B, F, G, H) and mean (D). Every symbol represents one mouse and lines are median values (C). Data was analyzed by two-tailed *t*-test (A, B, F, G, H) and one-way ANOVA with *t*-test correction (C, D). * *P* < 0.05, *** *P* < 0.001, **** *P* < 0.0001.

**Supplementary Fig. 2 Changes in gut microbiota due to high nucleosides diet and *L. plantarum* supplementation in mice. A** Rarefaction curves. Every curve represents one sample. Sequencing depth is sufficient to cover majority of gut microbiota when the slope of curve is close to zero. **B** Alpha-diversity characterized by Shanon, Chao, Ace, Shannoneven and Simpson indexes. Herein, microbial community diversity is positively correlated with Shanon index and negatively correlated with Simpson index. Microbial community richness is positively correlated with Chao and Ace indexes, and microbial community evenness is positively correlated with Shannoneven index. Three replicates are shown as circle. **C** Ratio of *Firmicutes* to *Bacteroidetes*. **D** Principal Component Analysis (PCA) of microbial communities. **E** Co-occurrence network integrating microbial OTUs. Circle (node) size corresponds to degree (number) that combined with other nodes. Yellow and bule edges represent positive and negative correlation, respectively, between two nodes. Edge (line) weight is proportional to correlation coefficient. OTUs with *p* value < 0.05 and correlation coefficient absolute value > 0.8. **F** Counts of two *Lactobacillus* species in feces. Scatter plot for correlation analysis between ND, HNS and HNS+LP mice. Dots indicate the counts of microbe (*X* axis) versus serum urate level (*Y* axis) (**G, H**) and versus other microbes (**I**). The dotted line shows the fitted result. Graphs show the Spearman rho correlation coefficient and one-tail *p* value. Graph shows the median with min to max (A), mean ± s.e.m. (C) and mean (F). Every point represents one mouse (C, D, F–I). Data was analyzed by one-way ANOVA with *t*-test correction (B, C) and one-tailed *t*-test (F). * *P* < 0.05.

**Supplementary Fig. 3 *L. plantarum* affecting the gut metabolites.** ^1^H NMR spectrum (**A)** and heatmap (**B**) of fecal metabolites. Panel **A** shows characteristic peaks of 35 fecal metabolites. Panel **B** indicates significant differences in their quantification between HNS and HNS+LP mice. Data was analyzed by two-tailed *t*-test. **P* < 0.05, ***P* < 0.01, ****P* < 0.001, *****P* < 0.0001. **C** Multi-omic network integrating microbial abundances (blue circles) with serum urate level (green diamond) and 20 fecal metabolites (red squares) that was significantly changed by *L. plantarum* supplementation in HNS mice. Node size and green edge of nodes correspond to degree (number) that combined with other nodes. Lines represents positive (red) and negative (blue) correlation, respectively, between two nodes. Edge (line) weight is proportional to correlation coefficient. Data are visible in Table S11 and S12.





**Supplementary Fig. 4** **Metagenomic functional annotation and classification of *L. plantarum* in KEGG (A) and in COG (B).** Different column colors correspond to functional classification of genes. Also, every initial of row indicates code of functional classification.

**Supplementary Fig. 5 Identification of production of inosine hydrolyzed by *L. plantarum*.** HPLC analysis confirmed degradation product of inosine was hypoxanthine, not urate and xanthine. MS analysis supproted from https://hmdb.ca/metabolites/HMDB0000157#spectra.

**Supplementary Fig. 6 Established Caco-2 cell monolayer for nucleosides and nucleobases transport.** Images of TEM (**A**), optical micrograph (**B**) and CLSM (**C**) of Caco-2 monolayer. **A** Blue and orange arrow presents microvillus and tight junction, respectively. **C** Blue (occludin) and orange (cell nucleus) fluorescence suggested the location of tight function between Caco-2 cells. The imagers were taken after 21-days incubation (A–C). Intracellular alkaline phosphatase activity (D), TEER (E) and uranine permeability (F) of Caco-2 cell line with 7-day (before) and 21-day (after) incubation. Graphs show the mean ± s.e.m. (D–F). Data was analyzed by two-tailed *t*-test. ****P* < 0.001, *****P* < 0.0001.


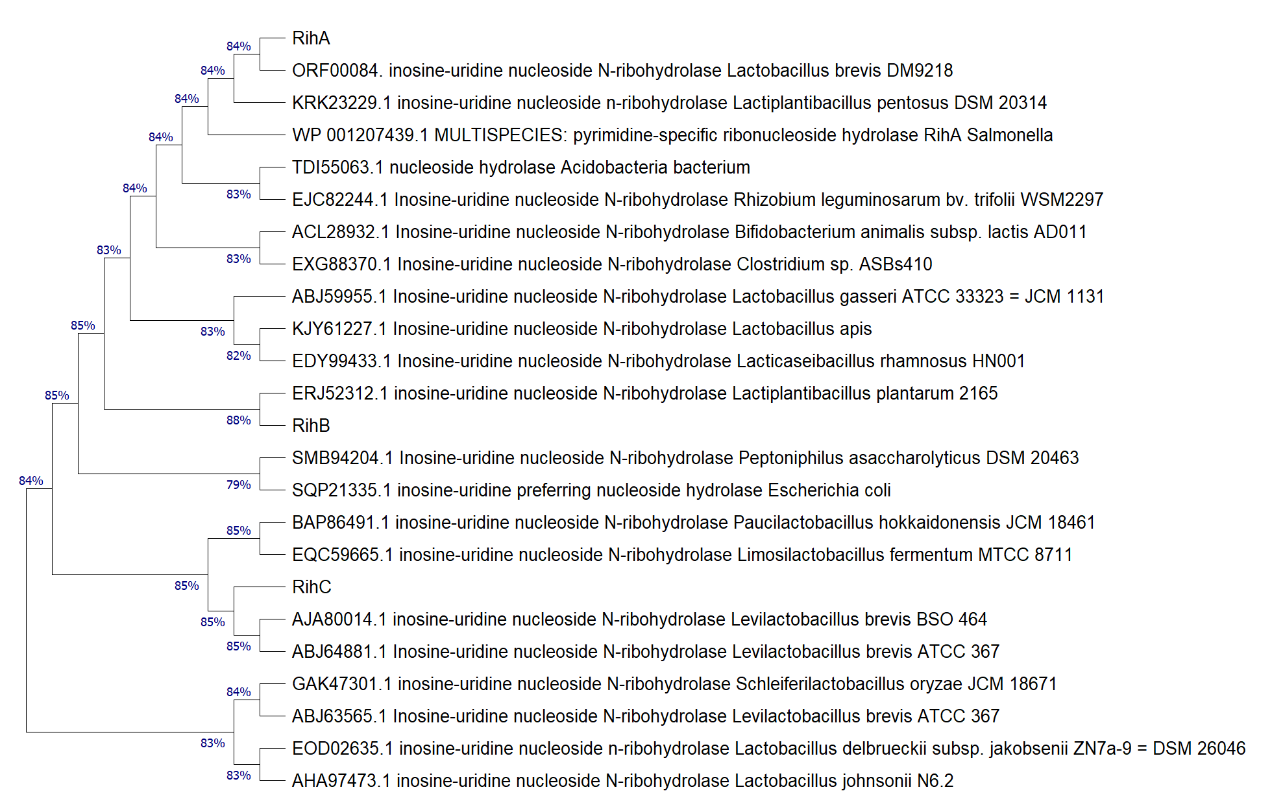


**Supplementary Fig. 7** **Gene sequences alignment of RihA–C from *L. plantarum* with other organisms.** Gene sequences of other organisms were provided by NCBI public dataset and visualized by MEGA software.
